# Simultaneous delivery of Paclitaxel and Bcl-2 siRNA via pH-Sensitive liposomal nanocarrier for the synergistic treatment of melanoma

**DOI:** 10.1038/srep35223

**Published:** 2016-10-27

**Authors:** Teegala Lakshminarayan Reddy, Koteswara Rao Garikapati, S. Gopal Reddy, B. V. Subba Reddy, J. S. Yadav, Utpal Bhadra, Manika Pal Bhadra

**Affiliations:** 1 Centre for Chemical Biology, CSIR-Indian Institute of Chemical Technology (IICT), Tarnaka, Hyderabad-500007, India; 2 Academy of Scientific and Innovative Research (AcSIR), Training and Development Complex, CSIR Campus, CSIR Road, Taramani, Chennai-600 113, India; 3 Centre for Semiochemicals, CSIR-Indian Institute of Chemical Technology (IICT), Tarnaka, Hyderabad-500007, India; 4 Functional Genomics and Gene Silencing Group, CSIR-Centre for Cellular and Molecular Biology (CCMB), Uppal Road, Hyderabad-500007, India

## Abstract

pH-sensitive drug carriers that are sensitive to the acidic (pH = ~6.5) microenvironments of tumor tissues have been primarily used as effective drug/gene/siRNA/microRNA carriers for releasing their payloads to tumor cells/tissues. Resistance to various drugs has become a big hurdle in systemic chemotherapy in cancer. Therefore delivery of chemotherapeutic agents and siRNA’s targeting anti apoptotic genes possess advantages to overcome the efflux pump mediated and anti apoptosis-related drug resistance. Here, we report the development of nanocarrier system prepared from kojic acid backbone-based cationic amphiphile containing endosomal pH-sensitive imidazole ring. This pH-sensitive liposomal nanocarrier effectively delivers anti-cancer drug (Paclitaxel; PTX) and siRNA (Bcl-2), and significantly inhibits cell proliferation and reduces tumor growth. Tumor inhibition response attributes to the synergistic effect of PTX potency and MDR reversing ability of Bcl-2 siRNA in the tumor supporting that kojic acid based liposomal pH-sensitive nanocarrier as efficient vehicle for systemic co-delivery of drugs and siRNA.

Chemotherapy is often considered the primary treatment for various cancers, although some serious dose-limiting side-effects are seen in most cases over a period of time^1^,^2^. Additionally, intrinsic or acquired multidrug resistance (MDR) is one amongst the major complications of chemotherapy. Research related to the development of better drugs towards broad spectrum of cancers have shown that microtubule depolymerising agents such as Paclitaxel (PTX), Docetaxel etc. have great potential to impede mitotic progression and induce caspase-mediated cell death^3^,^4^. Nonetheless, MDR decreases the drug accumulation inside the cell and increase the DNA damage repair mechanism, resulting in therapeutic inefficiency of any single agent^5^. Therefore, a combination of two or more therapeutic methods with a distinct mechanism of action needs to evolve as a promising medication in cancer therapy. The duo of small molecule and siRNA could potentially increase the therapeutic approach, target selectivity and effectively counteract drug resistance, thereby increasing therapeutic efficacy synergistically^6^.

Recently, a lot of attention has been paid in developing drug-delivery systems aiming at increased bio-availability, lower toxicity, higher efficiency and controlled release. Several materials including polymeric micelles^7^, dendrimers^8^, various nanoparticles^9^ and liposomes^10^ are being used in drug delivery systems. Research over a period of time has shown the delivery of small molecule therapeutics and its subsequent release in response to external stimuli such as temperature, light^11^,^12^, redox reagents^13^, ultrasound^10^, pH, enzymes, etc. Among these stimulation systems, the pH-responsive system is of particular interest for cancer therapy as both the tumor microenvironment (pH 6.8) and endosomes (pH 5.0) has more acidic pH compared to normal tissues (pH 7.4), thus enabling the carriers to release therapeutic agents in a pH-dependent manner^14^,^15^,^16^.

Earlier reports have demonstrated efficient delivery properties of pH-sensitive peptides, micelles^17^,^18^, pH-tunable endosomolytic oligomers, cationic polymers^19^,^20^, cationic liposomes^21^ and charge-conversional polyion complex^22^,^23^. Gene delivery into the cells via cationic lipoplexes (complex of liposomes and plasmid DNA) occur via endocytotic pathway after its localization within the endosomal compartment whereby the trapped DNA is released into the cytoplasm of the cells. Wolff and associates have introduced the use of endosomal pH-sensitive cationic lipids with imidazole head-groups. As the pKa of the weakly basic imidazole head- groups lie within the pH-range of endosomal environment (pH 5.5–6.5), the imidazole head-groups of cationic lipids acquire a proton (i.e. act as a proton sponge) whereby the lipoplexes enter the endosome compartments^24^. Such endosomal buffering by the imidazole head-groups results in osmotic swelling and subsequent release by rupture of the endosomes owing to the entry of hydrated chloride counterions. Further, studies have established the promising role of cationic amphiphiles with endosomal pH-sensitive histidine groups in increasing the drug delivery efficacy of cationic liposomes^15^.

In the present investigation, we have designed a novel pH-sensitive liposomal delivery system to co-deliver PTX and Bcl-2 siRNA into the tumor cells and mice models. We intended to overcome the multidrug resistance (by silencing Bcl-2 protein) and increase the therapeutic efficacy of PTX by co-delivering it with Bcl-2 siRNA. For this purpose, PTX and Bcl-2 entrapped cationic liposomal nanocarriers were formulated. The drug/gene complex has been extensively characterized by a range of biological studies together with apoptosis study, cell cycle analysis and cell proliferation assay. The systemic performance of lipo-PTX/siRNA has been depicted by pharmacokinetic study in experimental animals. Lastly, antitumor efficacy of lipo-PTX and lipo-PTX/siRNA has been performed in B16F10 cell bearing xenograft tumor model. Even though few reports have been published with reference to the combinational approach of anticancer drug and siRNA, the present investigation provides a detailed inclusive approach and also the attempt to counter multidrug resistance in melanoma tumor model.

## Results

### Design and Syntheses

Kojic acid, a natural compound produced by various microorganisms, especially by fungi of the *Aspergillus, Acetobacter and Penicillium genera* is a well known melanin inhibitor that possesses a whitening ability to melanocytes by producing a strong inhibition of tyrosinase^25^ that is readily accepted by normal melanocytes without causing toxicity. Here we have developed a scheme to functionalize kojic acid with imidazolium. Imidazole is known to have pH sensitivity and is also present in several drug moieties. We have modified cationic Kojic acid-imidazole (1-hexadecyl-3-((5-(hexadecyloxy)-4-oxo-4H-pyran-2-yl) methyl)-1H-imidazol-3-ium) lipid in four steps (Scheme-1, Supplementary Information). The detailed synthetic procedures of the molecule are described in the experimental section including ^1^HNMR and mass spectral data of the intermediates and final products. The structure of the final molecule was confirmed by ^1^HNMR, ^13^CNMR and mass spectroscopy. The purity of the final compound was confirmed by reversed phase HPLC using two mobile phases. Spectral data and HPLC profiles are provided in the spectral section (Supplementary Information).

### Formulation of PTX-siRNA encapsulated liposomes

Ever since cationic liposomes are being used for delivering siRNA, many attempts have been made to design a suitable liposomal carrier which can subsequently be used for complexation with the therapeutic siRNAs to result in siRNAplexes. But these types of electrostatic complexes are often associated with certain disadvantages of having bigger size, low stability and incomplete encapsulation of siRNA. This becomes a threat for the lipoplexes as its exposure to the serum nucleases causes degradation before getting delivered to the target organs, thereby becoming a major limiting issue for systemic delivery^26^. Therefore the challenge is to develop liposomal carriers capable of encapsulating siRNAs for targeted delivery under both *in vitro* and *in vivo* conditions.

We therefore underwent several formulations and after evaluating a number of possible combinations, the combination of Lipid: DOPE: DSPE-PEG-2000-NH_2_ in 1:1:0.05 molar ratios were found to be most efficient for effective siRNA and PTX encapsulation. When used in lipid:siRNA weight ratio of 25:1, encapsulation efficiency was about 90% (Table 1). In each case scrambled siRNA was used and each result produced is the average of triplicate experiments done on the same day. Preparation of stable liposomes with significant entrapment efficiency was not possible without incorporating lipid. Presumably the cationic character of the lipid led to interaction with the negative charges of siRNAs and thereby made the formulation stable. These liposome formulations showed more than 90% entrapment efficiency with both PTX and siRNA at 1/25 w/w ratio. Electrophoretic gel patterns confirmed that liposomes encapsulated siRNA with high entrapment efficiency (Figure S2).

### Physico-Chemical Characterization

**Figure 1:**
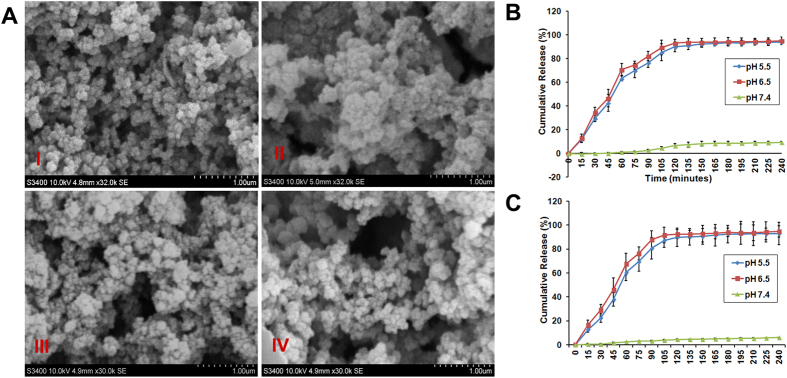
(**A**) Scanning Electron microscopic images of liposomes I) empty liposomes II) liposomes entrapped with only PTX III) Liposomes entrapped with only siRNA IV) Liposomes entrapped with both PTX and siRNA. Scale Bar is 1 μm. (**B**) Cumulative time course release profiles of siRNA from liposomes in pH dependant manner. (**C**) Cumulative time course release profiles of PTX from liposomes in pH dependant manner.

Hydrodynamic diameter of the liposomes containing siRNA, PTX were found to be within the size range of 110–130 nm. PTX/siRNA loaded liposomes were found to be slightly larger (136 ± 3) than that of empty liposomes (120 ± 10). Similarly surface potentials of PTX/siRNA loaded liposomes (34.5 ± 1.3) were lesser than that of empty liposomes (40.5 ± 1.2) (Table 1 and Figure S3). Scanning electron microscopic analysis of the liposomes showed sphere shaped structures with size ranging from 110–130 nm (Fig. 1A).

### pH dependant release of PTX and siRNA from liposomes

Release profile of siRNA from the liposomes was performed at different pH. Tumor microenvironment has a typical pH ranging from 6.5–6.9^6^. We have studied the release kinetics of siRNA from liposomes at different pH buffers starting from pH 5.5 to pH 7.4 at different time points by quantifying free siRNA and PTX in solution by performing Quant-iT ribogreen RNA quatification assay and spectrophotometry according to manufacturers protocol (Invitrogen, Carlsbad, CA, USA) respectively. These assays allowed us to measure the amount of free RNA and PTX in solution. We observed a highest release of siRNA and PTX (>90% at 6 h time interval) from liposomes at pH 6.5 and pH 5.5 respectively compared to rest of the pH conditions. In case of physiological pH (pH 7.4) the release was 10% (Fig. 1B,C). The pragmatic release pattern (Fig. 1B,C) clearly demonstrated that siRNA & PTX release from the liposomal formulations was most efficient at pH 6.5 and 5.5. Interestingly, the drug release pattern at pH values 6.5 & 5.5 showed a explode release in the first 1 h followed by a controlled release of siRNA & PTX over a period of 4 h (Fig. 1B,C) producing 90% of entrapped siRNA & PTX release during this time (Fig. 1B,C).

#### Protection of siRNA from Endonucleases

To prevent the digestion of siRNA by endonucleases, we performed a nuclease (RNase) protection assay, where samples of naked siRNA and siRNA entrapped liposomal formulations were analysed after RNase-A treatment. As expected, RNase-A digested the free siRNA showing no visibility in the lane 2 after ethidium bromide staining and liposomally entrapped siRNA was completely protected showing band at the well (lane 1) and after siRNA released from the liposome was slowly getting digested by RNase-A (lane-4). Here only free siRNA without RNase-A was used as negative control (lane-3) (Figure S2)^27^.

### Intracellular distribution and uptake of siRNA

**Figure 2:**
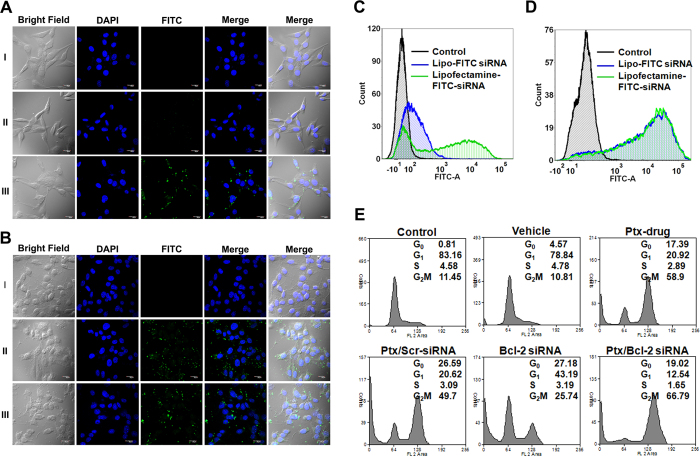
(**A**) Confocal Laser Scanning Microscopic (CLSM) images of intracellular distribution of FITC labeled siRNA entrapped liposomes in NIH/3T3 cells. (**B**) CLSM images showing intracellular distribution of FITC labeled siRNA entrapped liposomes in B16F10 cells. Nuclei counter stained with DAPI. (**C**) Flow cytometric analysis showing the cellular uptake of FITC labeled siRNA entrapped in liposomes in NIH/3T3 cells. (**D**) Flow cytometric analysis showing the cellular uptake of FITC labeled siRNA entrapped in liposomes in B16F10 cells. Scale 20 μm. **(E**) Flow cytometric analysis showing the synergistic effects of liposomes encapsulated Bcl-2 siRNA & PTX in cell cycle progression in melanoma tumor cells. B16F10 cells were treated with liposome containing PTX, liposome containing Bcl-2 siRNA, liposome containing both Bcl-2 siRNA & PTX and liposome containing scrambled siRNA and PTX drug. Both untreated and treated cells with vehicle served as controls.

To evaluate the cellular uptake of these liposomes, we transfected mouse murine melanoma cell line (B16F10) and mouse fibroblasts (NIH/3T3) grown on cover slips for 24 h at 37 °C with FITC labelled siRNA entrapped liposomes. Simultaneously in a parallel experiment, same cells grown on cover slips for 24 h at 37 °C were transfected with same siRNA using commercial transfecting agent Lipofectamine RNAiMAX which served as control. Both the cell lines were also transfected with naked siRNA that served as negative control. Upon visualization under confocal microscopy a clear uptake in the labeled siRNA was seen in B16F10 cells compared to naked siRNA treated cells under similar conditions. But, interestingly the uptake of FITC labelled siRNA entrapped liposomes was more pronounced in NIH/3T3 cells compared to the same cell line transfected with same siRNA using Lipofectamine showing that cells have their own specificity for uptake (Fig. 2A,B).

We next performed Flow Cytometry studies with the both the mentioned cell lines transfected either with FITC labelled siRNA entrapped liposomes or FITC labelled siRNA transfected using Lipofectamine. Flow cytometry data in B16F10 cells transfected with siRNA entrapped liposomal formulations showed almost equal uptake as observed under confocal microscope, whereas in case of NIH/3T3 cells the uptake was very less when compared with cells transfected with same siRNA using lipofectamine (Fig. 2C,D).

### Effect of co-delivery on cell proliferation

To observe the effect of co-delivery of siRNA and PTX, we performed cell cycle analysis on B16F10 cells 24 h after treatment with liposome formulations containing both PTX and Bcl-2 siRNA. We also performed cell cycle analysis on B16F10 cells after treating them for 24 h with either Bcl-2 siRNA or PTX. Simultaneously studies were also performed under same parameter using the empty vehicle to see whether there was any toxic effect of the vehicle itself or not. Normal growing cells without any treatment was taken as control to see the cell cycle profile. Cells treated with only liposome formulation (vehicle) did not show any toxicity and the cell cycle profile was similar to control (Fig. 2E), illustrating non toxic and biocompatibility of the formulation. Cells treated with PTX drug and lipo-PTX/scrambled siRNA showed an arrest of 50% at G2M phase, confirming the activity of PTX as a mitotic inhibitor that arrests cells in G2M phase. Whereas cells treated with lipo-Bcl-2 siRNA showed an arrest of 20% cell population at sub-G1 phase, indicating that inhibition of Bcl-2 induces apoptosis, as Bcl-2 is an anti apoptotic gene. But cells treated with both PTX and Bcl-2 siRNA entrapped liposomal formulations showed a clear arrest of 20% cell population at subG1 and 66% population at G2M phase indicating a clear delivery of both Bcl-2 siRNA and PTX (Fig. 2E).

### *In Vitro* Apoptotic analysis

**Figure 3:**
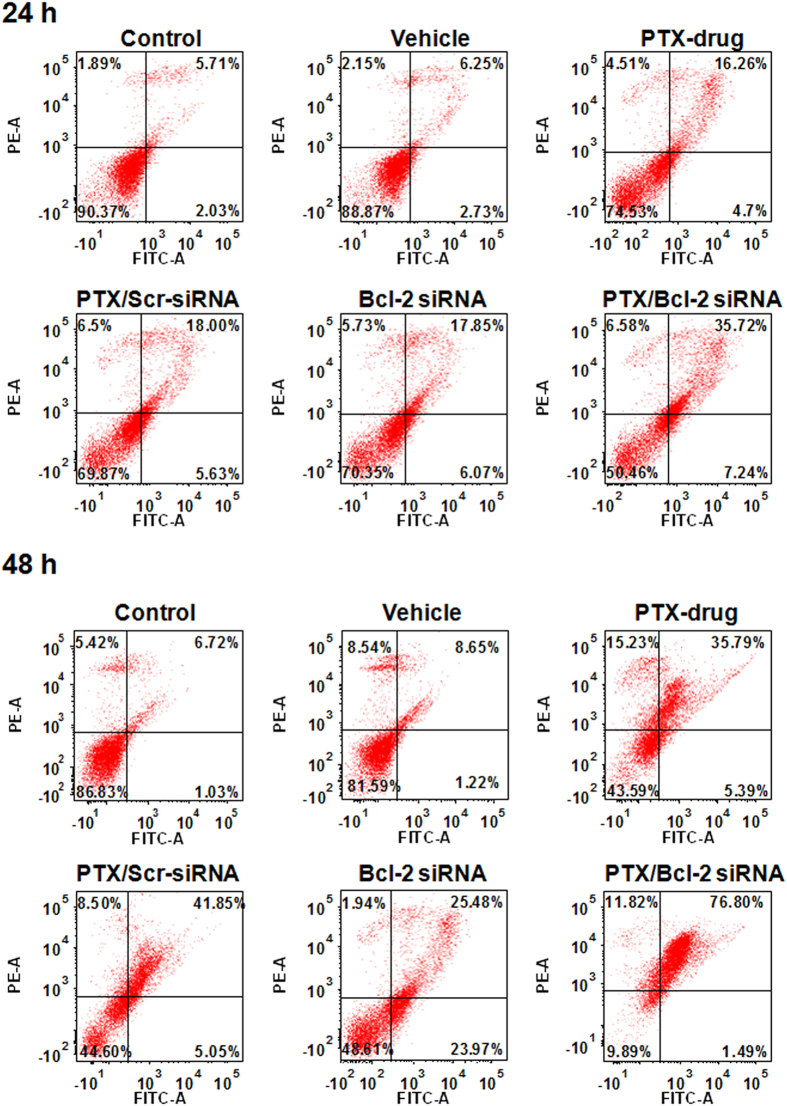
Synergistic effects of Liposome encapsulated Bcl-2 siRNA & PTX in inducing apoptosis in melanoma tumor cells in time dependant manner. B16F10 cells were treated with Liposome containing PTX, Liposome containing Bcl-2 siRNA, Liposome containing both Bcl-2 siRNA & PTX and Liposome containing scrambled siRNA and PTX drug. Both untreated and treated cells with vehicle were stained with Annexin V-FITC and propidium iodide (PI) for flow cytometric analysis. The horizontal and vertical axes represent cells labeled with FITC-Annexin V and PI, respectively in the dot plot. Dots in the upper right quadrant represent late apoptotic cells (positive for both Annexin V and PI).

To perceive the effect of co-delivery of PTX and Bcl-2 siRNA on apoptosis we performed Annnexin-V-FITC assay at two different time points i.e. 24 h and 48 h cells treated with either empty vehicle, or PTX drug or lipo-PTX/Bcl-2 siRNA or lipo-PTX/scrambled siRNA. Cells treated with empty vehicle did not show any apoptosis similar to untreated controls at both time points (Fig. 3) confirming that liposome formulation produces no effect on normal cell cycle progression. But, in all other treated cells apoptotic population was more at 48 h than that of 24 h. At 24 h cells treated with either PTX drug or lipo-PTX/scrambled siRNA or lipo-Bcl-2 siRNA liposomes showed 18% apoptotic population, where as lipo-PTX/Bcl-2 siRNA showed 36% apoptosis (Fig. 3). 48 h post transfection, all combination of liposomes followed similar pattern except in case of co delivery of lipo-PTX/Bcl-2 siRNA wherein a clear increase of apoptosis to 76% was observed. This is probably due to knockdown of Bcl-2 by its siRNA which sensitizes the cells towards drug action and reverse multidrug resistance in cells (Fig. 3).

### Tumor growth inhibition studies

**Figure 4:**
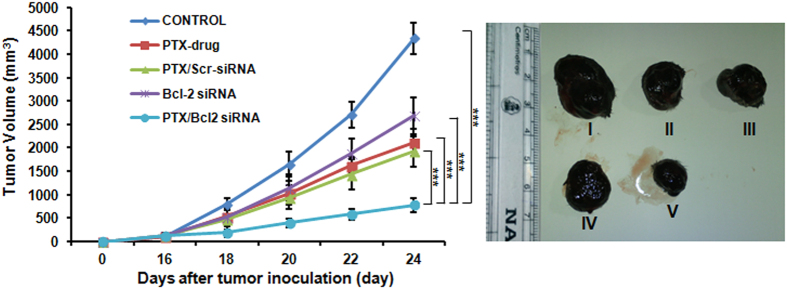
*In vivo* studies showing the inhibition in tumor growth in mice upon injection with liposomal formulations containing both PTX & Bcl-2 siRNA. (**A**) Relative tumor growth inhibition upon injecting liposomal formulations with either PTX or Bcl-2 siRNA or liposomal formulation containing both PTX & Scrambled siRNA. (**B**) Representative tumor sizes in each group on day 24 post tumor inoculation. Mice administered with PBS (I); Mice administered with PTX drug (II); Mice administered with liposome containing PTX & Scrambled siRNA (III); Mice administered with liposomal Bcl-2 siRNA (IV); Mice administered with liposome containing both PTX & Bcl-2 siRNA (V). (***P < 0.001).

In order to confirm the efficacy of co-delivery with our lipo formulation and its therapeutic efficacy at the animal level, we conducted antitumor study in C57BL/6J xenograft B16F10 tumor model. As expected, injecting the animals with saline did not produce inhibition of tumor growth and rapidly reached maximum tumor volume at the end of day 24. Injecting PTX alone at a concentration of 0.9 mg/kg body weight produced a regression of tumor to a small extent showing inefficiency in delivering the drug for controlling its proliferation. The lipo-PTX/Scrambled siRNA exhibited relatively better tumor growth inhibition as compared to free PTX. Similarly, lipo-Bcl-2 siRNA injection alone was unable to reduce the tumor volume to a considerable extent. Whereas, co-delivery of lipo-PTX/Bcl-2 siRNA showed significant (p < 0.001) inhibition of tumor growth compared with other experimental groups (Fig. 4). This could be due to the synergistic inhibitory effect of PTX and Bcl-2 siRNA on the tumor proliferation. The significant tumor growth inhibition was due to the therapeutic combination of siRNA that further increased the chemotherapeutic efficacy of PTX. This data clearly supports the fact that combination or co-delivery of drug and gene is an ideal platform for tumor therapy (Fig. 4).

### Analyses of therapeutic effects *in vitro* and *in vivo*

**Figure 5:**
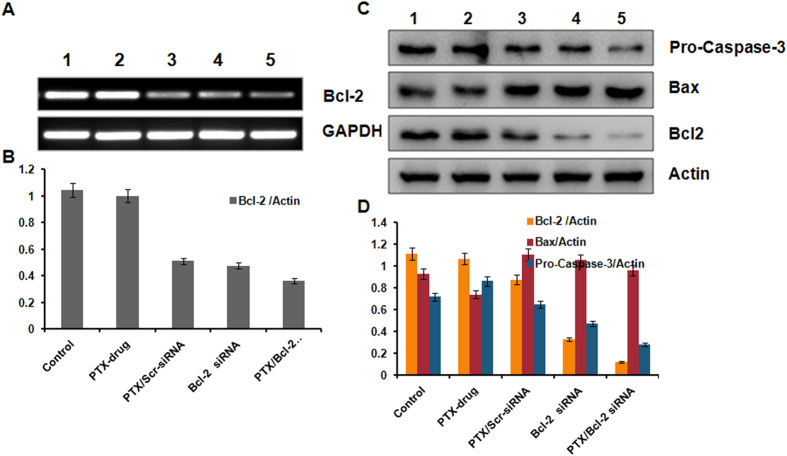
mRNA and Protein expressions of indicated genes and proteins involved in proliferation and apoptosis as measured by RT-PCR and Western blotting. (**A**) RT-PCR studies showing the knockdown of Bcl-2 mRNA. (**B**) Bars represent relative Bcl-2 gene expression normalized to GAPDH (housekeeping gene as gel loading control) from same sample with SD. (**C**) Western blot analysis showing the elevated protein expression levels of Bcl-2, Bax & Pro caspase-3. Lane 1, Untreated Cells; lane 2, Cells treated with PTX drug; lane 3, Cells treated with liposome containing PTX & Scrambled siRNA; lane 4 Cells treated with liposomal Bcl-2 siRNA; lane 5, Cells treated with liposome containing both PTX & Bcl-2 siRNA. (**D**) Bars represent relative protein expression normalized to β-actin (housekeeping gene as gel loading control) from same sample with SD.

Anti-apoptotic Bcl-2 gene was selected as the target for RNA interference in the present study. As the ratio of the anti-apoptotic Bcl-2 protein to pro-apoptotic Bax protein determines the chemo resistance in cancer cells^28^, the effect of treatment on pro-apoptotic Bax gene was also evaluated. We therefore performed quantitative RT PCR and western blot analysis both *in vitro* and *in vivo* to evaluate the level of expression of these genes at both transcriptional and translational level to understand the molecular mechanism of action of its therapeutic effects. B16F10 cells were incubated for 24 h with liposomes carrying either PTX or Bcl-2 siRNA or complexed with both. RNA and proteins were isolated for qPCR as well as Western blot studies. qPCR studies with RNA isolated from B16F10 cells treated with liposomes carrying Bcl-2 siRNA or complexed with both Bcl-2 siRNA and PTX showed a clear down regulation of Bcl-2 mRNA demonstrating an effective delivery into B16F10 cells. PCR studies showed a clear variation in the expression of Bcl-2 gene in response to the PTX concentration though initial studies showed that the expression level of Bcl-2 mRNA was stable in PTX drug treated group. In other words, downregulation of Bcl-2 mRNA was also observed in the PTX/Scrambled-siRNA group. This is probably due to the reason that reports have shown that PTX inhibits the expression of Bcl-2. Excitingly, the co-delivered Bcl-2 siRNA/PTX effectively suppressed Bcl-2 expression to significant levels compared to untreated controls or treated under conditions mentioned (Fig. 5A,B).

In line with the RT-PCR results, Bcl-2 protein was significantly not changed in PTX drug group but clearly suppressed in PTX/Scr-siRNA and Bcl-2 siRNA groups. More importantly, the PTX/Bcl-2 siRNA group exhibited the lowest Bcl-2 protein expression and significant over expression of Bax. We also checked the protein expression of Pro-caspase-3 under all treatment conditions as Pro-caspase-3 is a key molecular indicator for cells undergoing apoptosis. Pro-caspase-3 protein was also down regulated (Fig. 5C,D). Consequently, the lowest level of Pro-caspase-3 protein was detected in PTX/Bcl-2 siRNA group indicating activation of cell apoptosis.

**Figure 6:**
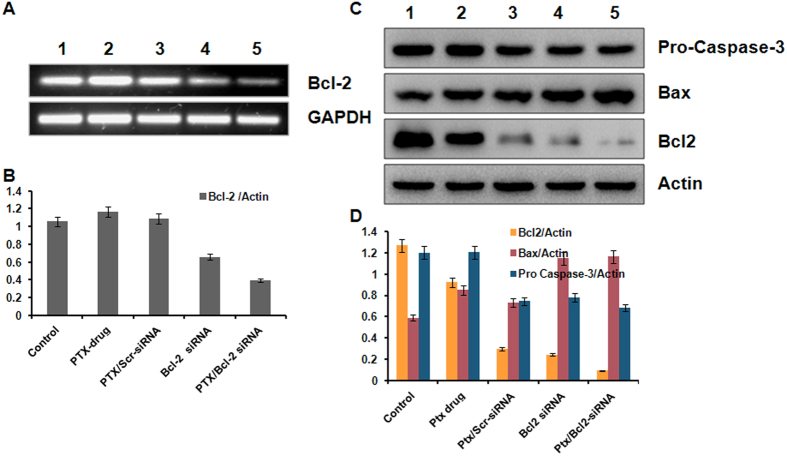
*In vivo* studies showing synergestic effect in inhibiting expressions of proliferation and anti-apoptosis related genes. Liposome formulations containing both PTX and Bcl-2 siRNA were injected into B16F10 tumor bearing mice and the expression of proliferation and anti-apoptosis related genes was measured after isolating proteins from the tumor samples. (**A**) RT-PCR analysis showing the knockdown of Bcl-2 mRNA. (**B**) Bars represent relative Bcl-2 gene expression normalized to GAPDH (housekeeping gene as gel loading control) from same sample with SD. (**C**) Western blot analysis showing elevated expression of Bcl-2, Bax & Pro caspase-3 proteins. (**D**) Bars represent relative protein expression normalized to β-actin (housekeeping gene as gel loading control) from same sample with SD. Lane 1, Mice administered with saline; lane 2, Mice administered with PTX drug; lane 3, Mice administered with liposome containing PTX & Scrambled siRNA; lane 4, Mice administered with liposomal Bcl-2 siRNA; lane 5, Mice administered with liposome containing both PTX & Bcl-2 siRNA.

We further performed similar RT PCR and western blot studies under *in vivo* conditions for the same genes under *in vitro* conditions after isolating RNA and proteins from tumor samples untreated or under treatment with PTX or Bcl-2 siRNA or in various other combinations either encapsulated or not encapsulated with liposomes (Fig. 6C,D). The same trend of expression was found in apoptotic proteins under *in vivo* conditions (Fig. 6C,D). A clear knock down of Bcl-2 gene was observed at both mRNA and protein levels (Fig. 6A,B) in tumor samples administered with liposome formulations.

### Immunohistochemical studies

**Figure 7:**
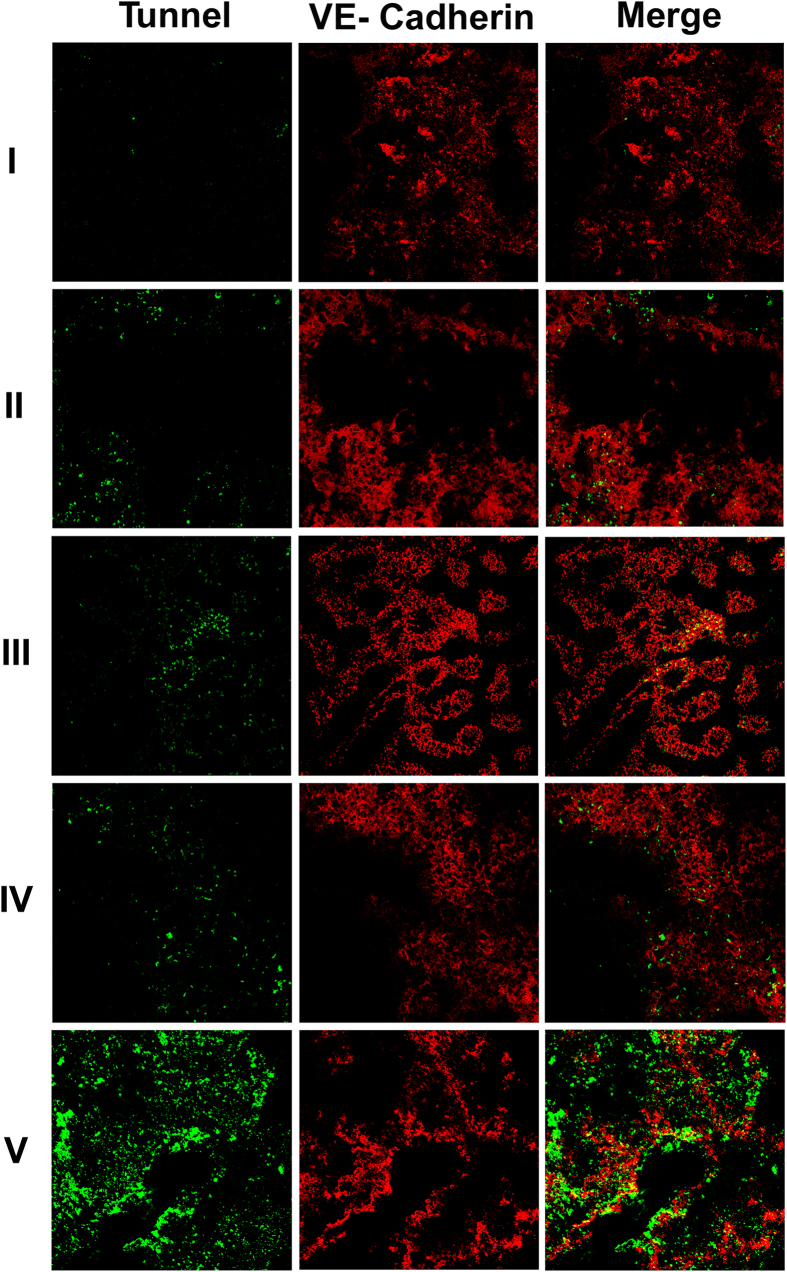
Tumor growth inhibition by intratumoral administration of the co-formulations of PTX & Bcl-2 siRNA in liposomes is mediated through apoptosis of tumor endothelial cells. B16F10 melanoma cell inoculated mice were randomly sorted into five groups (n = 5 for each group). (**I**) Mice administered with saline. (**II**) Mice administered with PTX drug. (**III**) Mice administered with liposomes containing both PTX & Scrambled siRNA, (**IV)**. Mice administered liposomes containing Bcl-2 siRNA, (**V)**. Mice administered with liposomes containing both PTX & Bcl-2 siRNA. On day 24 post tumor inoculation, the fixed tumor cryosections from each group were immunostained with anti-VE-cadherin antibodies (marker of tumor endothelial cells) and TUNEL assay kit (markers for apoptotic cells, green). All the images were taken at 10x magnification.

Toward examining the possibility that co-delivery of PTX Bcl-2 siRNA encapsulated liposomes may lead to apoptosis of the tumor endothelial cells, fixed tumor cryosections prepared from tumor bearing mice treated with PTX drug alone and Bcl-2 siRNA formulated liposomes were immunostained with tumor endothelial cell marker VE-cadherin and TUNEL following manufacturer’s protocol (Clontech Laboratories, Mountain View, CA, USA). Significant co-localization of the TUNEL and VE-cadherin (markers of tumor endothelial cells) positive cells in tumor cryosections was observed in mice administered with above formulations (Fig. 7). No such localization was seen in mice administered with PBS confirming the observation (Saline) (Fig. 7).

## Discussion

In clinical cancer treatments, chemotherapy is currently facing tough challenges including insufficient tumor targeting, systemic side effects and drug resistances, posing difficulty in developing anti cancer drugs^29^,^30^,^31^. Though recent advancements in nanomedicinal technology have brought tremendous hope for addressing these problems, *in vivo* application of anti-cancer drugs is still a big hurdle. Delivery vehicles mediated by nanocarrier systems has numerous advantages over conventional chemotherapy, such as lessened side effects, increased bioavailability, long blood circulation and passive tumor accumulation merely due to the nanosize^32^. To date, advanced nanocarriers with molecular targeting function^33^, bioenvironmental stimuli-responsiveness^34^,^35^ and drug/gene co-delivery capacity^36^,^37^ are regularly designed and developed. These nanocarriers can significantly improve the therapeutic efficacy of many anti-cancer drugs via enhanced tumor targeting and/or better control of the drug release behavior. However, most nanoscale systems developed for drug delivery purpose are still far from being ideal due to their intrinsic shortages. In particular, these problems become even more intractable in developing nanocarriers accommodating the co-delivery of anti-cancer drugs and nucleic acids since it is much more difficult to get small particle size in this case. We showed that two therapeutic agents-loaded in pH-responsive liposomal formulation (PTX/Bcl-2 siRNA) accumulated much more effectively in tumor tissue. Here we demonstrated the pH-responsive biocompatible co-delivery of siRNA and PTX with our liposomal formulation. We have designed our lipid with Kojic acid as backbone moiety, which is readily accepted by the skin. The formulation has not shown any toxicity. For stimuli responsive release we have attached imidazole, which is known to show pH-responsive release. Since the pKa of weakly basic imadazole is around 6, it gets protonated within the acidic endosomal environment (pH 5.5–6.5) producing a natural pH response release^38^. The enhanced ability of delivering PTX/Bcl-2 at the tumor microenvironment potentially may support the low-dose systemic chemotherapy with low side effects.

However, despite its tumor-killing effect, clinical PTX applications are associated with severe side effects including nausea, vomiting and bone marrow depression^39^. Therefore, it is highly desirable to attain maximum therapeutic effect with a low dose of PTX^10^. We found that, when B16F10 cells were treated with low-dose of PTX (0.642 μg/mL), not only the caspase dependent cell apoptosis was induced but also the expression of anti-apoptotic Bcl-2 protein was down regulated. Apparently, low-dose PTX treatment led to anti-apoptotic response as reported in other studies as well^40^,^41^, which highlights the great significance of silencing Bcl-2 gene during PTX chemotherapy. In the present study, using our pH-sensitive liposomal nanocarrier system we successfully delivered two therapeutic agents PTX and Bcl-2 siRNA, simultaneously into cancer cells in time dependant manner with no toxicity. Consequently, the PTX-inducible drug resistance in cells was effectively reversed by parallel silencing the Bcl-2 gene during the process of chemotherapy. Thus, the synergistic actions of PTX and Bcl-2 siRNA on cancer cells and in tumor model resulted in the significantly improved anti-cancer effect of PTX^42^.

## Conclusion

In summary, present study investigated the simultaneous delivery of siRNA and chemotherapeutic drug by pH-sensitive liposomal delivery system demonstrating the potential of co-delivery of PTX and Bcl-2 siRNA to enhance the chemotherapeutic efficacy in tumor cell line. The lipo-PTX/siRNA exhibited sustained release kinetics and effectively inhibited the cell proliferation. Co-delivery to overcome the Bcl-2 mediated drug resistance during chemotherapy, enabling low-dose yet effective PTX treatment of cancer. Additionally, delivery system is biocompatible and has not showed any toxicity. *In vivo* antitumor study exhibited a significant tumor regression profile. The favourable tumor inhibition response was attributed to the synergistic effect of PTX potency and MDR reversing ability of siRNA in the tumor.

## Methods

### Syntheses

Synthesis of target modified cationic Kojic acid (1-hexadecyl-3-((5-(hexadecyloxy)-4-oxo-4H-pyran-2-yl) methyl)-1H-imidazol-3-ium) lipid was done from imidazole and kojic acid. Imidazole was treated with hexadecanyl bromide in the presence of NaH at 0 °C to produce N-alkylated product 2. O-alkylation of enolic hydroxyl group of kojic acid with hexadecyl bromide in the presence of K_2_CO_3_ in acetone under reflux conditions afforded the compound 4, which was converted into bromo derivative 5 using CBr_4_ and TPP at 0 °C. Finally the coupling of compound 2 and 5 in acetonitrile under reflux conditions gave the target compound 6 in good yield and all the structures of the compounds were characterized using NMR, Mass, IR spectroscopy respectively. Purity of the compound was checked with HPLC.

### Preparation of Liposomes

Cationic liposomal nanoparticles were prepared by thin-film hydration technique as reported^43^. Briefly, Cationic Kojic acid based lipid, 1,2-dioleoyl-sn-glycero-3-phosphoethanolamine (DOPE), 1,2-distearoyl-sn-glycero-3-phosphoethanolamine-N-[amino(polyethylene glycol)-2000] (DSPE-PEG (2000)-NH2) (1:1:0.05) molar ratio were dissolved in chloroform–methanol (3:1, v/v) in glass vial. Paclitaxel (1/25 w/w) was added before the thin-film process along with the lipids. Solvent was removed with a thin flow of moisture free nitrogen gas and the dried lipid film was kept under high vacuum for 8 h. The lipid film was hydrated (allowed to swell) for 12 h in RNase free water containing siRNA and protamine sulfate (14:1, w/w) such that the total lipid:siRNA ratio (w/w) became 25:1. The mixture was vortexed for 5 min followed by bath sonication for 2 min. The resulting liposomal suspension was subjected to seven freeze/thaw cycles by alternately placing the sample vial in a dry ice bath and 37 °C water bath. The mixture was further transferred to an Amicon Ultra, 30 kDa, (Chemicon, Temecula, CA, USA) and the unentrapped free siRNAs were removed by centrifugation at 10000 rpm for 30 min.

### Entrapment Efficiency

Entrapment efficiency of liposomal formulations was calculated from the total amount of drug added versus amount of drug entrapped in the liposomes. Dual loaded liposomes briefly centrifuged at a speed of 12000 rpm for 10 min. Filtrate was collected and analyzed for unentrapped drug by HPLC method. The mobile phase consisting of methanol:water (70:30) was set at 1 ml/min and the effluents were monitored at 227 nm. A standard curve of PTX was plotted.


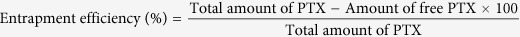


The siRNA entrapment efficiencies were determined using scrambled siRNAs. Briefly dual loaded complex was centrifuged at a speed of 12000 rpm for 10 min. Free siRNA in the solution was quantified by using by Quant-iT Ribogreen RNA assay kit as per manufacturers protocol. The liposomal samples were diluted with RNase free water so that the siRNA concentrations of the diluted samples were within the linear range of the standard graph constructed with known concentrations of the siRNAs. The amounts of entrapped siRNAs were estimated by measuring fluorescence intensities (Varioskan-Flash microplate fluorescence reader, Thermo Instruments, USA) of the liposomal solutions lysed with 0.1% SDS using 480 nm and 520 nm as excitation and emission wavelengths, respectively. Encapsulation efficiency was calculated from the formula:


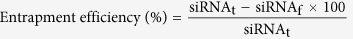


Where siRNA_t_ is the known amount of siRNA used in preparing liposomal formulation and siRNA_f_ is the amount of siRNA recovered after liposomal disruption with SDS. All the measurements were done in triplicate.

### siRNA entrapment and Gel retardation assay

1 μg of scrambled siRNA in was entrapped inside the liposomes by freeze thaw method. On the other hand liposomes incubated with 1 μg siRNA scrambled siRNA at room temperature for 20 minutes before loading onto the gel. Above mentioned samples and the equivalent amount of naked siRNA was separately loaded on to the gel. The electrophoretic mobility of twas visualized using an ultraviolet illuminator with ethidium bromide staining after electrophoresis on 2% (w/v) agarose gel for 20 minutes at 80 V in TAE buffer (40 mM Tris-HCl, 1% v/v acetic acid, 1 mM EDTA).

### Particle size distribution and zeta potential

The liposome solutions were suitably diluted with double distilled water such that mean count rate was around 300 kcps to analyze the particle size distribution and zeta potential using dynamic light scattering (DLS) method. Malvern Zetasizer (Malvern, UK) was used to determine the DLS characteristics. All measurements were performed at a fixed angle of 90° at 25 °C room temperature. The results were expressed as the size ± SD.

### pH Triggered Release (*In Vitro* Release study)

*In vitro* release of siRNA from lipo-siRNA and lipo-PTX/siRNA was monitored by quantifying the amount of free siRNA. In this study, 1 ml of NP dispersion was taken in 1.5 mL tube, centrifuged at 12000 rpm for 10 min and supernatant collected in new tube. Pellet was dispersed in different pH buffers (pH 5.5, 6.5, 7.4) and the whole set up was placed in an automated shaker maintained at 100 rpm at 37 °C. At predetermined time intervals, release media was collected and replaced with equal amount of fresh media. The released siRNA was quantified using Quant-iT Ribogreen RNA assay kit as mentioned above.

Release of PTX from lipo-PTX and lipo-PTX/siRNA was monitored by quantifying the amount of free PTX. The released PTX was quantified spectrophotometrically at a wavelength of 227 nm by using uv sensitive cuvettes (Eppendorf, Hamburg, Germany).

### Apoptosis detection assay

2 × 10^5^ B16F10 cells were plated in each well of 6-well plate and allowed to grow for 24 h after which they were treated with PTX drug, lipo-PTX/Scrambled siRNA, lipo- Bcl-2 siRNA and lipo-PTX/Bcl-2 siRNA and allowed to incubate for 24 h and 48 h. ApoAlert™ Annexin V-FITC Apoptosis Kit was used to measure apoptotic cells by flow cytometry according to the manufacturer’s instruction (Clontech Laboratories, Inc. CA, USA). Cells collected by trypsinization were washed twice with ice cold Dulbecco’s phosphate buffered saline (DPBS) and resuspended in 200 μL of 1X binding buffer containing 5 μL Annexin V and 10 μL Propidium iodide for 15 min at room temperature in the dark. Apoptosis of cells was measured on a Beckman Coulter flow cytometer (DakoCytomation, Inc. CA, USA). At least 20,000 gated events were acquired from each sample. Results are expressed as the percentage of apoptotic cells (PI and Annexin V positive) in the gated cell population.

### Tumor inhibition study

All animal experiments were carried out in accordance with the Institute Ethical Committee (CSIR-IICT) approval. The antitumor efficacy of free PTX, lipo-PTX, lipo-Bcl-2 siRNA and lipo-PTX/Bcl-2 siRNA was investigated on 6-weeks C57BL/6 J mice (each weighing 20–22 g). Approximately ~1.5 × 10^5^ B16F10 cells in 250 μL Hank’s buffer salt solution were injected subcutaneously with a syringe attached with a 30 gauge needle in the right flank of the mice on day 0. On day 14 when tumor volume reached approximately 100 mm^3^, mice were randomly sorted into groups and each group (n = 5) was administered with respective formulations at a dose of 1 mg/kg body weight on days 15, 17, 19, 21 and 23. Subsequently, tumor volume was measured at specified time using vernier caliper in two dimensions. Tumor volume (V) was measured by the formula: V = (L × W^2^)/2, wherein length (L) is the longest diameter and width (W) is the shortest diameter perpendicular to length. Simultaneously body weight of individual mice was noted to interpret the toxicity or safety profile of each formulation. The tumor mass was surgically removed at the end of the study period and compared. Among the photographs of the representative tumors were also taken on day when the tumor was surgically removed. Results represent the means ± SD (for n = 5).

### *In Vivo* apoptotic analysis (Immunohistochemical studies)

Mice were sacrificed 24 h post last day injections and tumors were excised. 5–10 micron thickness tumor cryosections were obtained using cryostat instrument (Leica) and weer mounted on a charged glass slide. The slides were fixed in 4% methanol-free formaldehyde in 1X PBS. Slides were washed twice with PBS. Thereafter tissue permeabilization was achieved by administration of Triton X-100 solution (0.2% in 1X PBS) for 5 min. Sub-sequently, tissue was blocked with a 1% BSA solution for 60 min followed by incubation with anti-VE-Cadherin (endothelial cells, red) (Santa Cruz Biotechnology, CA, USA) antibody (1:100) at room temperature for 2 h. The slides were washed three times each for 5 min with PBST and incubated with Cy-3-conjugated anti-goat secondary anti-body (Jackson Immuno Research Laboratories Inc, PA, USA) for 45 mins. Thrice subsequent washes with PBST solution were performed. The slides were further performed with TUNEL assay kit (Clontech Laboratories Inc, USA) for apoptotic tumor endothelial cells as per manufacturer’s protocol. The cover slips were mounted on to slides with mounting media. Slides were observed under confocal microscope (Olympus FV1000) and images were taken with the support of the flow view version 1.7c software program. The stained slides were observed under confocal laser Scanning microscope (Olympus FV1000) and images were taken with the support of the flow view version 1.7c software program and Cy3 (red) channel for VE-cadherin and in green field for TUNEL staining.

### Statistical Analysis

Statistical Analysis was performed using the graph-pad prism software to evaluate the significant difference between the control and treated samples. All variables were tested in three independent experiments. The results were reported as mean ± SD. *indicates p < 0.05, **indicates p < 0.01 and ***indicates p < 0.001.

## Additional Information

**How to cite this article**: Reddy, T. L. *et al*. Simultaneous delivery of Paclitaxel and Bcl-2 siRNA via pH-Sensitive liposomal nanocarrier for the synergistic treatment of melanoma. *Sci. Rep.* **6**, 35223; doi: 10.1038/srep35223 (2016).

**Publisher’s note:** Springer Nature remains neutral with regard to jurisdictional claims in published maps and institutional affiliations.

## Supplementary Material

Supplementary Information

## Figures and Tables

**Table 1:** Size and zeta potential measurements of siRNA and PTX encapsulated liposomes.

Liposomal formulations	Hydrodynamic diameter (nm)	Zeta potential (mV)	siRNA entrapment efficiencies (%) (1/25)	PTX entrapment efficiencies (%) (1/25)
Empty Liposomes	120 ± 10	40.5 ± 1.2	NA	NA
PTX entrapped Liposomes	127 ± 6	37.7 ± 0.7	NA	92.7 ± 5
siRNA entrapped Liposomes	128 ± 8.4	33.2 ± 0.3	92.6 ± 4.5	NA
PTX/siRNA entrapped Liposomes	136 ± 3	34.5 ± 1.3	94 ± 5	91.2 ± 5.2

Entrapment efficiency of liposomes with respect to siRNA and PTX.
