# Supplementary material for: Simultaneous delivery of Paclitaxel and Bcl-2 siRNA via pH-Sensitive liposomal nanocarrier for the synergistic treatment of melanoma
Source: Sci Rep. 2016 Oct 27;6:35223. doi: 10.1038/srep35223 (PMC5081533; doi:10.1038/srep35223)
Supplement: Supplementary Information [file srep35223-s1.pdf]

Simultaneous delivery of Paclitaxel and Bcl-2 siRNA via pH sensitive liposomal nanocarrier for the synergistic treatment of melanoma.

Teegala Lakshminarayan Reddy,[1,4] Koteswara Rao Garikapati,[1,4] S. Gopal Reddy,[2] B. V. Subba Reddy,[2] JS Yadav,[2] Utpal Bhadra,[3] and Manika Pal Bhadra.[1,4]*

Supplementary Methods

Materials

Kojic acid, Bromohexadacane, Imidazole, protamine sulfate (fraction X from Salmon), Paclitaxel and cell culture media (DMEM) Phosphate buffer saline were purchased from Sigma Aldrich, (St. Louis, MO, USA). Unless otherwise stated all reagents were purchased from Sigma-Aldrich (St. Louis, MO, USA) without further purification. 1,2-dioleoyl-*sn*-glycero-3-phosphoethanolamine (DOPE), 1,2-distearoyl-*sn*-glycero-3-phosphoethanolamine-N-[amino(polyethylene glycol)-2000] (ammonium salt) (DSPE-PEG (2000)-NH_2) procured from Avanti Polar Lipids (Alabaster, AL, USA). B16F10 (mouse murine melanoma cells) and NIH/3T3 (Mouse Fibroblasts) cell lines were procured from the ATCC (Manassas,VA, USA). Cells were grown in Dulbecco's modified Eagle's medium (DMEM) with 10% FBS (South American Origin, Gibco, Carlsbad, CA, USA) and 1% penicillin-streptomycin-kanamycin (Gibco, Carlsbad, CA, USA) at 37 °C in a humidified atomosphere containing 5% CO_2 in air. 6-8 week old C57BL/6J mice (each weighing 20-22 g) were purchased from the National Institute of Nutrition, Hyderabad, India. All the in vivo experiments were performed in accordance with the Institutional Bio-Safety and Ethical Committee Guidelines using an approved animal protocol. ^{1}H NMR spectra were recorded on a Varian FT 300 MHz Spectrometer. Column chromatography was performed with silica gel (Acme Synthetic Chemicals, India, 60–120 mesh). SuperScript II, a first strand cDNA Synthesis Kit purchased from Invitrogen Corporation, (Carlsbad, CA, USA), PCR Master Mix 2×, Annexin V-FITC apoptosis detection kit and TUNEL assay kit purchased from Clontech Laboratories Inc.(Palo alto, CA, USA). All the siRNAs were procured from Dharmacon (Lafayette, CO, USA) in deprotected, desalted and annealed forms (Table S1). Anti-Bcl-2, VE-cadherin and Anti Bax antibodies were purchased from Santa Cruz Biotechnology (Santa Cruz, CA, USA). Anti-Procaspase-3 antibody was purchased from Imgenex Biotechnology (Bhubaneswar, Odisha,

India) and anti β-Actin antibody was purchased from Abcam (Cambridge, MA, USA). HRP conjugated goat anti-rabbit and goat anti mouse secondary antibodies were purchased from Santa Cruz Biotechnology (Santa Cruz, CA). Cy-3 conjugated anti-rabbit secondary antibody was purchased from Jackson Laboratories (West grove, PA, USA).

Particle size distribution and zeta potential

The hydrodynamic sizes of liposomal formulations were analyzed by dynamic light scattering (DLS) performed on a DynaPro Nano DLS system (Wyatt Technologies, Santa Barbara, CA, USA). A Zetasizer (Malvern, UK) was used to determine the zeta potential of the siRNA encapsulated liposomes. All measurements were performed at a fixed angle of 90 ^{0} at room temperature (25°C). The results were expressed as the size ± standard deviation and zeta potential ± standard deviation.

siRNA entrapment and Gel retardation assay

1 µg of scrambled siRNA in was entrapped inside the liposomes by freeze thaw method. On the other hand was liposomes incubated with 1 µg siRNA scrambled siRNA at room temperature for 20 minutes before loading onto the gel. Above mentioned samples and the equivalent amount of naked siRNA was separately loaded on to the gel. The electrophoretic mobility of twas visualized using an ultraviolet illuminator with ethidium bromide staining after electrophoresis on 2% (w/v) agarose gel for 20 minutes at 80 V in TAE buffer (40 mM Tris-HCl, 1% v/v acetic acid, 1 mM EDTA).

RNase protection assay

To prevent digestion of siRNA by endonucleases, we performed a nuclease protection assay, where samples of naked siRNA and the siRNA entrapped liposomal formulations and electrostatic lipoplexes were treated with RNase-A for 30 minutes. The samples were then treated with 8 U of RNase-A inhibitor at 37°C for 15 minutes and run on 2% agarose gel for 20 minutes at 80 V in TAE buffer (40 mM Tris-HCl, 1% v/v acetic acid, 1 mM EDTA). The gel was visualized under an ultraviolet illuminator after staining with ethidium bromide.[1]

Morphology

The morphological examination of empty Liposomes, lipo-PTX, lipo-siRNA and lipo-PTX/ siRNA was performed using SEM, Hitachi S-3400N Tokyo, Japan. Liquid sample was

placed on a coverslip, dried and coated with gold before observing and analyzing under scanning electron microscopy.

Cellular uptake by flow cytometric analysis

The B16F10 and NIH/3T3 cells were seeded at a density of 2 x 10^5 cells/6-well plate and allowed to adhere for 24 h. The cells were exposed to lipo-PTX/FITC-siRNA and incubated for 4 h. The cells were washed twice with PBS, trypsinized, collected and re-suspended in PBS. The amount of cellular uptake was confirmed by flow cytometry (Becton Dickinson, Sunnyvale, CA).

Confocal microscopy

Confocal Laser Scanning microscopic (CSLM) analysis was carried out to observe the uptake of liposomes within the cells. B16F10 and NIH/3T3 cells were grown on coverslips for 24 h, followed by transfection with FITC-labelled siRNA entrapped liposomal nanparticles for 4 h. The cells were washed with PBS buffer and fixed with 4% paraformaldehyde (PFA) for 20 min. The cells were then mounted with vectashield mounting media (Vector Laboratories, Inc, Burlingame, CA, USA) and observed under FV1000 confocal laser scanning microscope (FV1000, Olympus, Japan).

Cell cycle analysis by flow cytometry

B16F10 cells were seeded at a density of 2 x 10^5 cells/6 well plate and allowed to adhere for 24 h. Thereafter the cells were treated with liposomes and incubated for 24 h at 37 °C in a standard incubator. The cells were trypsinized, harvested, and centrifuged using a microcentrifuge at 1500 rpm for 4 min. The cell pellets were washed twice with PBS buffer and fixed in 75% ethanol solution at 4 °C followed by brief centrifugation, and washed with PBS. They were again resuspended in PBS containing 5 mg/mL PI and 50 mg/mL deoxyribonuclease-free ribonuclease A. The suspension was incubated in dark for 25 min and analysed for cell cycle patterns using flow cytometry.

RT-PCR analysis

Total RNA was extracted using RNeasy mini kit (Qiagen, Valencia, CA, USA) and reverse transcribed into cDNA using superscript II reverse transcriptase (Invitrogen, Carlsbad, CA. USA). PCR was carried out with specific primers for GAPDH, Bcl-2 (Table S1) in Eppendorf thermal cycler. The products were electrophoresed on agarose gel (1%) containing ethidium

bromide and visualized under U V light. The signal intensity of respective bands was measured by means of the Quantity One version 4.1.1 software using BIORAD image analysis system (Hercules, CA, USA).

Western blot analysis:

To assay changes in apoptotic protein levels, after co-delivery with liposomes in B16F10 cells, both treated and untreated cells were collected and lysed in RIPA lysis buffer (Sigma-Aldrich, St. Louis, MO, USA) . Cell lysate was collected by centrifugation.

To observe the expression of apoptotic proteins both before and after co-delivery of liposomal samples in tumor, 1mg of tumor tissue was collected, washed with PBS and homogenised in RIPA lysis buffer. Protein lysate was collected by centrifugation.

In both *in vitro* and *in vivo* experimental condition, total protein concentration was determined using a Bradford micro protein Assay protocol (Sigma–Aldrich, St. Louis, MO, USA). For performing western blot analysis, 30 μg of total protein from each sample were loaded on each well of 10% sodium dodecyl sulphate-polyacrylamide gel (SDS-PAGE)and electrophoresed. The proteins were transferred to polyvinylidene difluoride (PVDF) membranes and blocked with 5% non fat dry milk (Santa Cruz Biotechnology, Santacruz, CA, USA) in Tris-buffered saline with Tween 20 (TBST) for 1 hour. Blots were hybridized overnight at 4 °C with primary monoclonal anti-Bcl-2, anti-Bax (Santa Cruz Biotechnology, Santacruz, CA, USA), anti-β-actin (Abcam, Cambridge, MA, USA) and anti-Procaspase-3 (Imgenex, Bhubaneswar, Odisha, India) antibodies, followed by incubation with HRP-conjugated anti rabbit and anti mouse secondary antibodies (Santa Cruz Biotechnology, Santacruz, CA, USA). Level of proteins were detected using Crescendo chemi luminiscent detection reagents (Millipore, Billerica, MA, USA) and visualized with a Bio-Rad chemi Doc XRS+ imaging system with Image Lab acquisition and analysis software (Bio Rad, CA, USA).

Reference:

1 Tagalakis, A. D., He, L., Saraiva, L., Gustafsson, K. T. & Hart, S. L. Receptor-targeted liposome-peptide nanocomplexes for siRNA delivery. Biomaterials 32, 6302-6315, doi:DOI 10.1016/j.biomaterials.2011.05.022 (2011).

Supplementary Tables

Table S1: Oligo sequences (siRNA and Primers) used in the study.

S.No	Name	Sequence
1	Bcl-2 siRNA-Sense	5′-GUG CCU AUC UGG GCC ACA ATT
2	Bcl-2 siRNA-Anti Sense	5’-UUG UGG CCC AGA UAG GCA CTT
3	Bcl-2 Forward Primer (Human)	5′-GGG GAG AAG GT G TTC ATT CA -3′
4	Bcl-2 Reverse Primer (Human)	5′-CAA CTC TTT TCC TCC CAC CA-3′
5	Bcl-2 Forward Primer (Mouse)	5′-GCCTTCTTTGAGTTCGGTGG-3′
6	Bcl-2 Reverse Primer (Mouse)	5′-CAGCCTCCGTTATCCTGGAT-3′
7	GAPDH Forward Primer	5′-GGGAAGGTGAAGGTCGGAGT-3′
8	GAPDH Reverse Primer	5′-TTGAGGTCAATGAAGGGGTCA-3′

Supplementary Figures

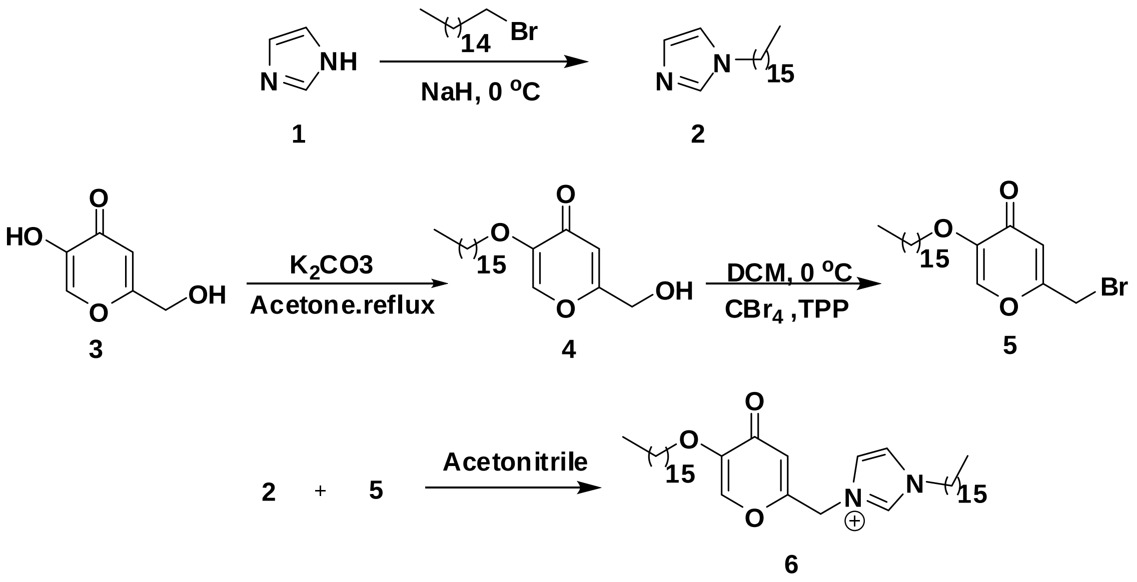

Scheme 1: synthetic scheme of the target cationic lipid.

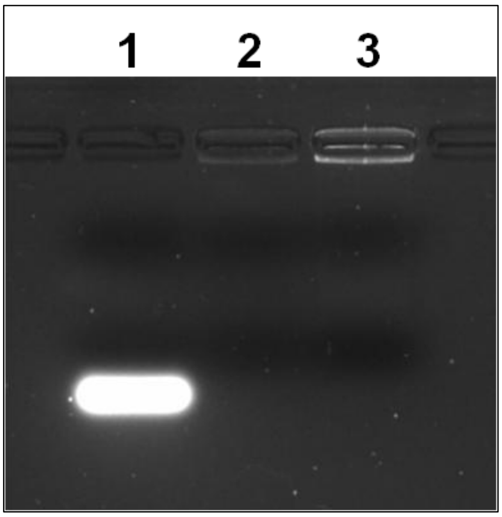

Figure S1: Relative electrophoretic mobilities of free siRNA (lane 1), siRNA encapsulated within the liposomes (lane 2) and electrostatic complex (lane 3). In each case 1 µg of non-silencing siRNA was used.

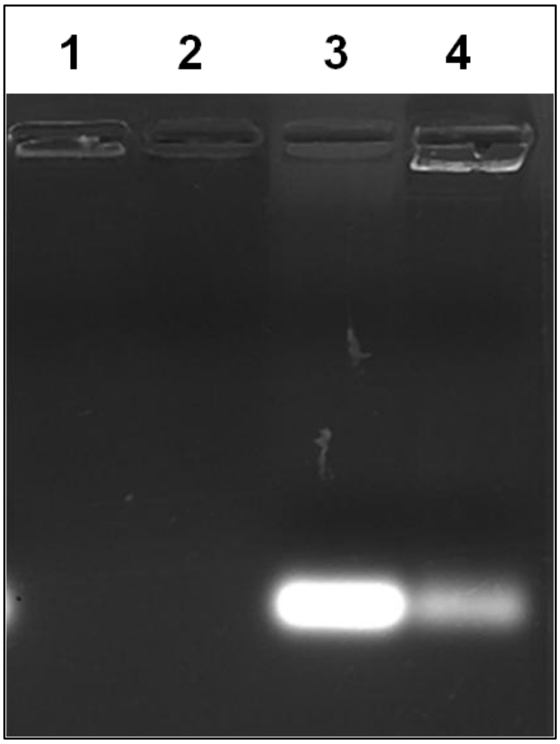

Figure S2: RNase protection assay. Degradation of siRNA exposed to RNase, lipo-siRNA complexes and compared with free siRNA**.** siRNA encapsulated within the liposomes + RNAse-A (lane 1), Free siRNA + RNase-A (lane 2), Only siRNA (lane 3), siRNA encapsulated within the liposomes after slow release + RNase-A (lane 4). In each case 1 µg of non-silencing siRNA was used.

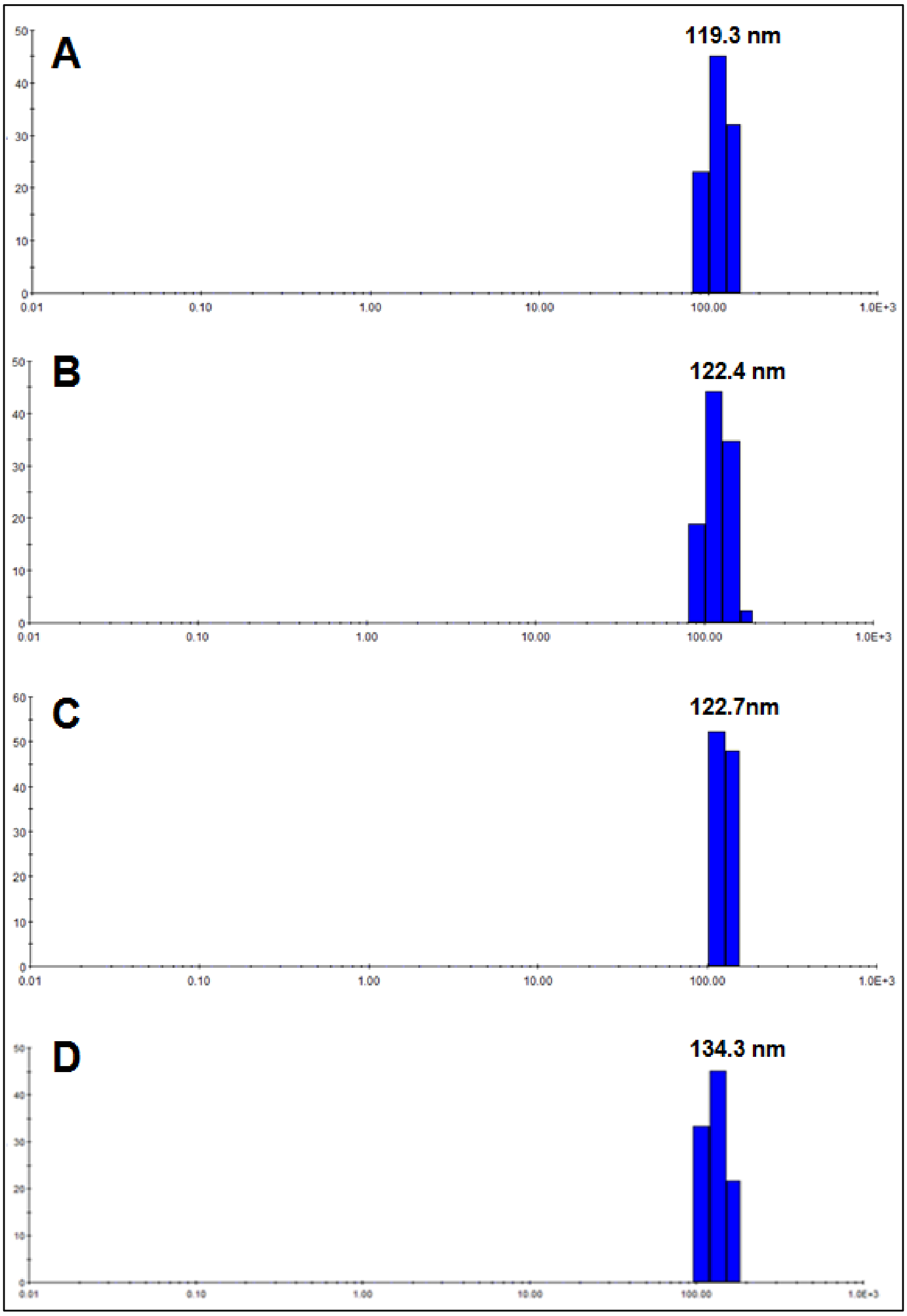

Figure S3: Dynamic Light Scattering images of liposomes A) empty liposomes B) liposomes entrapped with only PTX C) Liposomes entrapped with only siRNA D) Liposomes entrapped with both PTX and siRNA.

Synthetic methods and Spectral data of prepared compounds:

1-hexadecyl-1H-imidazole (Compound-2)

1-bromohexadecane (3.2mmol) was added drop wise to a stirred solution of imidazole (3.2mmol) and NaH (3.41mmol) in THF (20mL) at 0 ^{0}C. Reaction mixture was maintained at ambient temperature for 30 min. After completion of reaction, quenched with water and extracted with ethyl acetate (2x50) and dried over sodium sulphate.

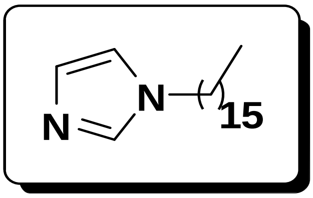

Solid, m.p.124-126 °C; ^{1}H NMR (300 MHz, $CDCl_3$): δ 7.51 (s, 1H), 7.06 (s, 1H), 6.90 (s, 1H), 3.93 (t, *J*= 6.9 Hz, 2H), 1.77 (m, 2H), 1.26 (m, 26H), 0.88 (t, J=6.4Hz, 3H) ppm; ^{13}C NMR (75 MHz, $CDCl_3$): δ 136.9, 128.8, 118.7, 47.1, 31.8, 31.0, 29.6, 29.5, 29.4, 29.3, 29.0, 26.4, 22.6, 14.0 ppm.

IR (KBr): ν_{max} 3445, 2923, 2854, 1631, 1463, 1025, 616 cm^{-1}.
MS (EI): *m/z* ($[M]^+$): 348; HRMS (EI): *m/z* calcd for $C_{22}H_{20}O_2S$: 348.1184; found: 348.1182.

5-(hexadecyloxy)-2-(hydroxymethyl)-4H-pyran-4-one (Compound-4)

Kojic acid (7.04mmol) was dissolved in dry DMF and added K_2CO_3(8.8mmol,) after 30 mins 1-bromohexadecane was added dropwise to the above solution. Heated the reaction mixture overnight and extracted the organic solvent with ethyl acetate and dried over sodium sulphate and purified by column chromatography.

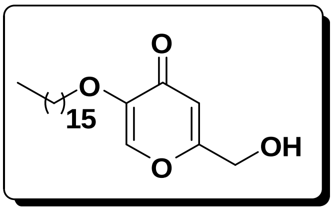

Solid, m.p.124-126 °C; ^{1}H NMR (300 MHz, $CDCl_3$): δ 7.55 (s, 1H), 6.51 (s, 1H), 4.48 (s, 2H), 3.84 (t, *J*= 6.7 Hz, 2H), 2.85 (s, 1H), 1.81 (m, 2H), 1.42 (m, 2H), 1.26 (m, 26H), 0.88 (t, *J*= 6.8 Hz, 3H) ppm; ^{13}C NMR (75 MHz, $CDCl_3$): δ 174.7, 166.8, 147.8, 139.0, 111.8, 69.7, 60.9, 31.9, 29.6, 29.5, 29.3, 28.9, 25.7, 22.6,14.1 ppm ;

IR (KBr): ν_{max} 3454, 3196, 3079, 2915, 2848, 1649, 1617, 1468, 1364, 1275, 1219, 1152, 1068, 1017, 994, 944, 870, 811, 748, 717, 543 cm^{-1}.

MS (EI): *m/z* ($[M]^+$): 348; HRMS (EI): *m/z* calcd for $C_{22}H_{20}O_2S$: 348.1184; found: 348.1182.

1-hexadecyl-3-((5-(hexadecyloxy)-4-oxo-4H-pyran-2-yl)methyl)-1H-imidazol-3-ium (Compoud-6)

CBr_4 (3.3mmol) and triphenyl phosphene (3.3mmol) was added to 5-(hexadecyloxy)-2-(hydroxymethyl)-4H-pyran-4-one (2.76mmol) dissolved in dichloromethane at 0 ^{0}C. Reaction was maintained at ambient temperature for 1 h. Then solvent was evaporated and the crude product obtained was used without further purification.

The above prepared compound (2.3 mmol) and 1-hexadecyl-1H-pyrrole (2.3mmol) were taken in to acetonitrile and refluxed for 3 h. Reaction mixture was cooled to RT and washed with ethyl acetate for three times to obtained pure product as off white coloured solid.

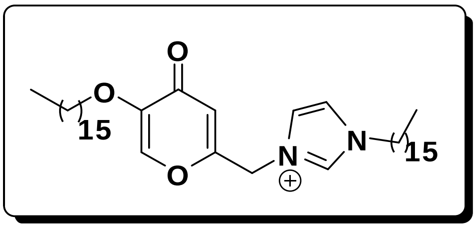

Solid, m.p.124-126 °C; ^{1}H NMR (300 MHz, $CDCl_3$): δ 9.91 (s, 1H), 7.84 (s, 1H), 7.75 (s, 1H), 7.71 (m, 1H), 6.61 (s, 1H), 4.28 (t, *J*= 7.5 Hz, 2H), 3.84 (t, *J*= 6.6 Hz, 2H), 2.59 (m, 2H), 1.84 (m, 4H), 1.26 (m, 52H), 0.88 (t, *J*= 6.2 Hz 6H) ppm; ^{13}C NMR (75 MHz, $CDCl_3$): δ 172.2, 157.4, 146.8, 138.4, 135.9, 122.9, 121.7, 114.1, 68.3, 48.9, 48.3, 30.6, 28.8, 28.3, 28.1, 28.0, 27.7, 24.9, 24.5, 21.4, 13.0 ppm.

IR (KBr): ν_{max} 3449, 2924, 2855, 1630, 1042, 618, 543 cm^{-1}.

MS (EI): *m/z* ($[M]^+$): 348; HRMS (EI): *m/z* calcd for $C_{22}H_{20}O_2S$: 348.1184; found: 348.1182.

NMR Spectra of the compounds:

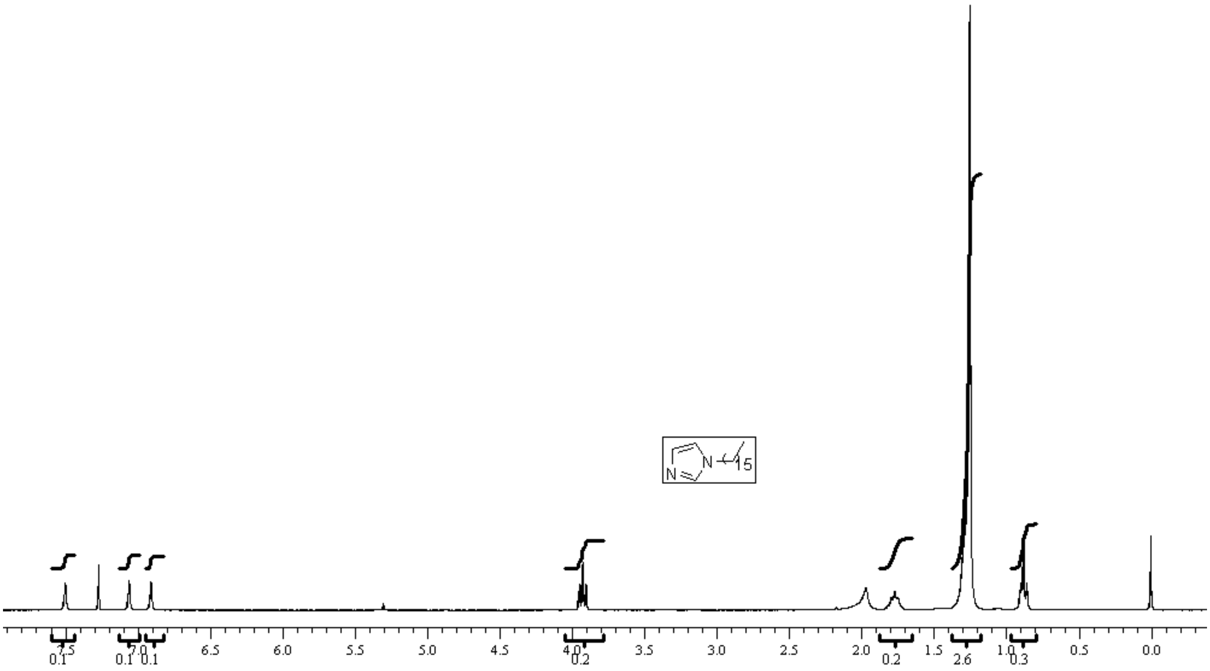

^{1}H NMR of 1-hexadecyl-1H-imidazole

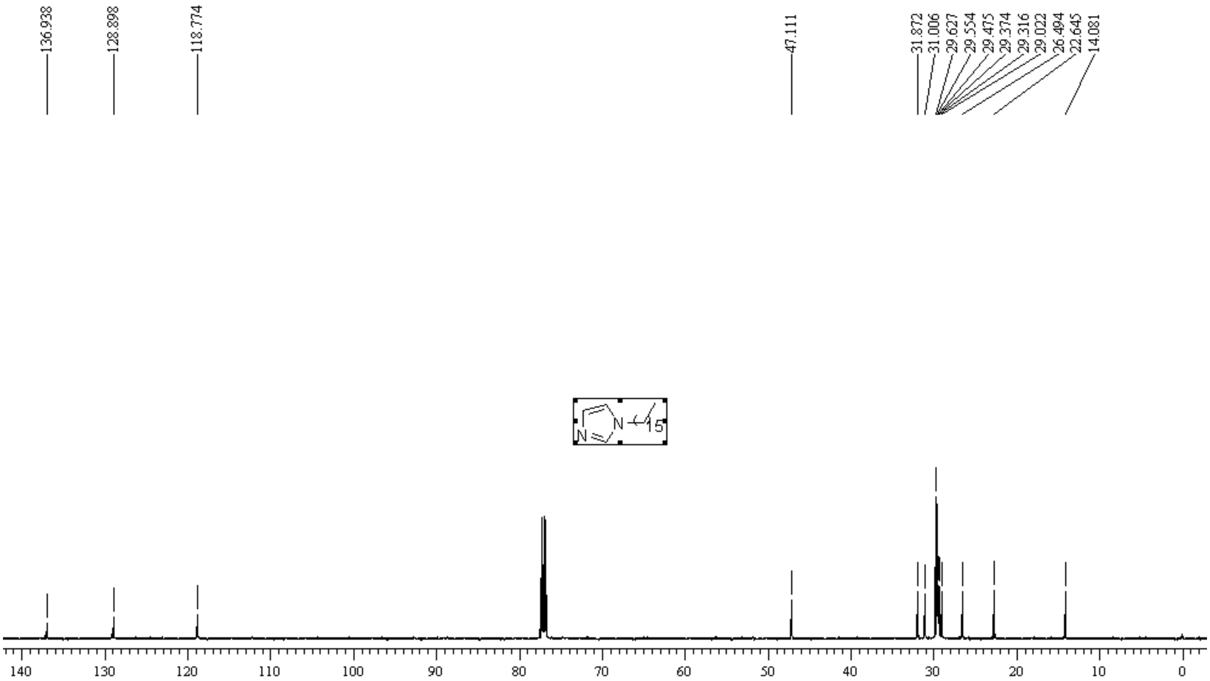

^{13}C NMR of 1-hexadecyl-1H-imidazole

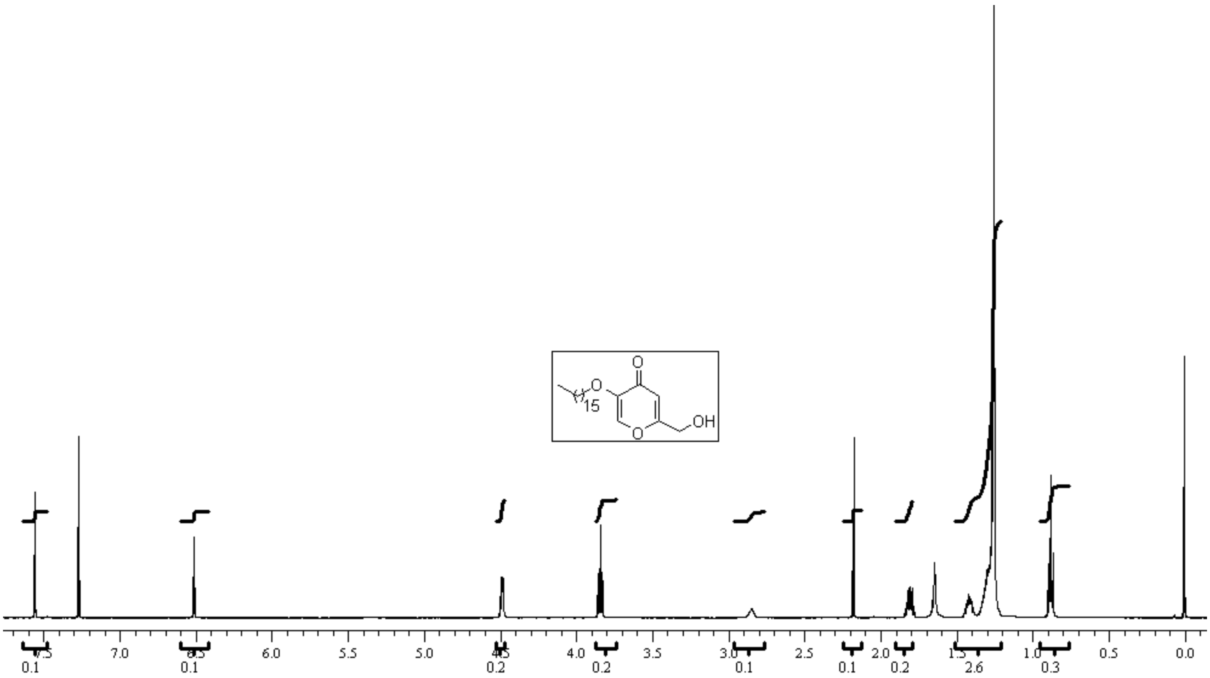

^{1}H NMR of 5-(hexadecyloxy)-2-(hydroxymethyl)-4H-pyran-4-one

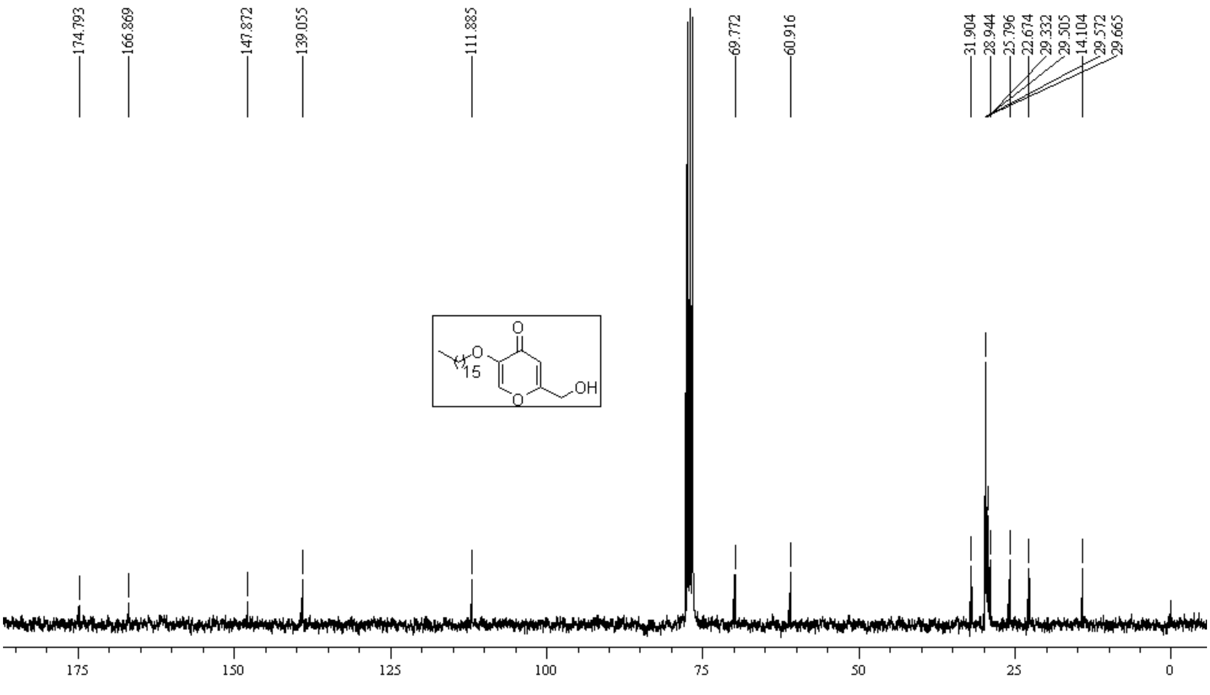

^{13}C NMR of 5-(hexadecyloxy)-2-(hydroxymethyl)-4H-pyran-4-one

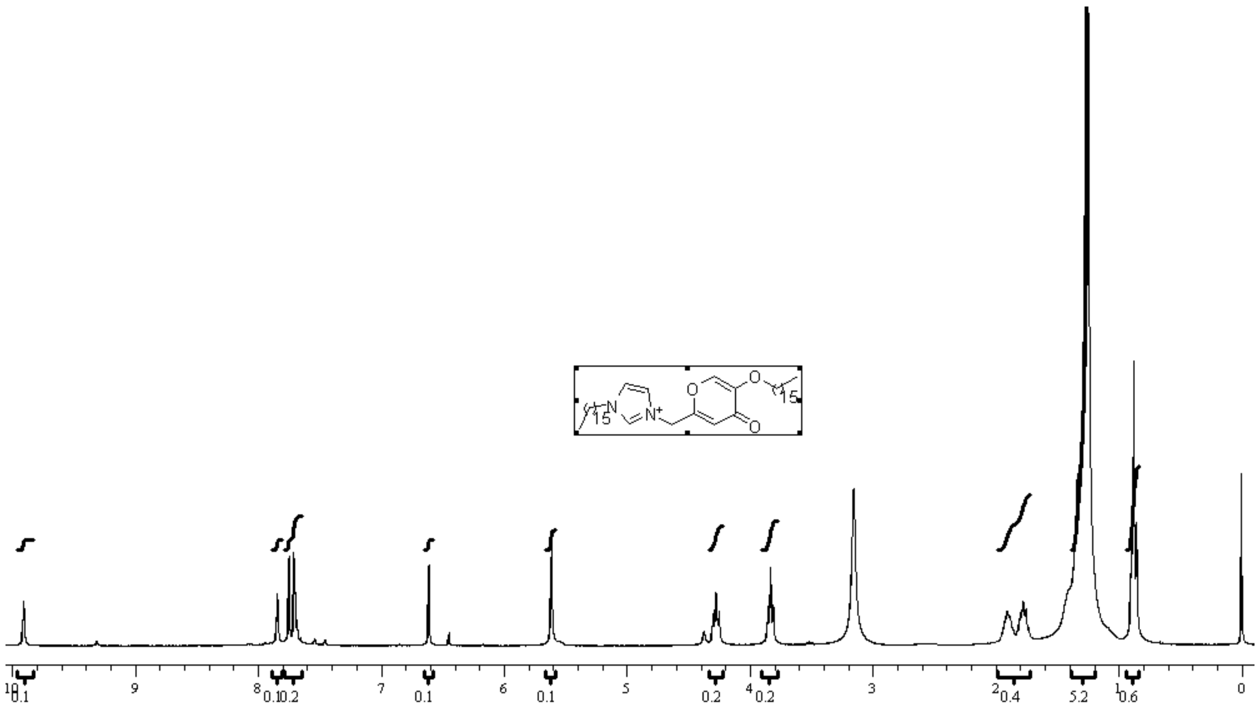

^{1}H NMR of 1-hexadecyl-3-((5-(hexadecyloxy)-4-oxo-4H-pyran-2-yl)methyl)-1H-imidazol-3-ium

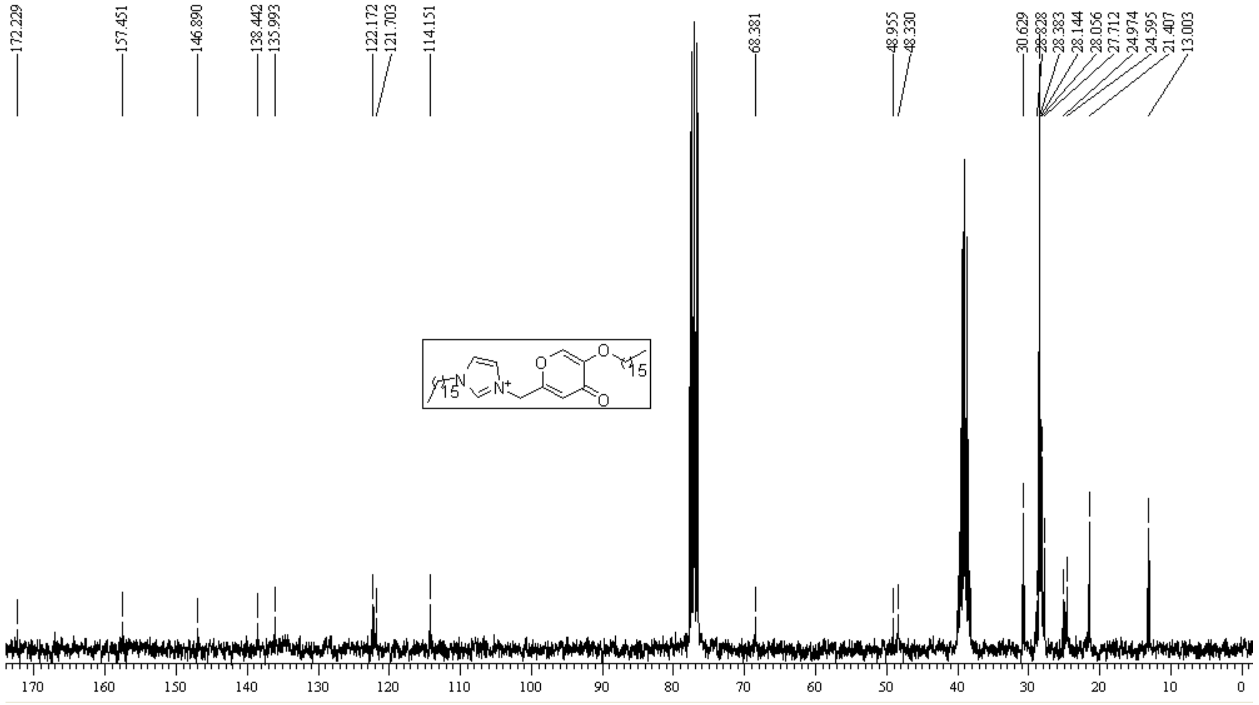

^{13}C NMR of 1-hexadecyl-3-((5-(hexadecyloxy)-4-oxo-4H-pyran-2-yl)methyl)-1H-imidazol-3-ium

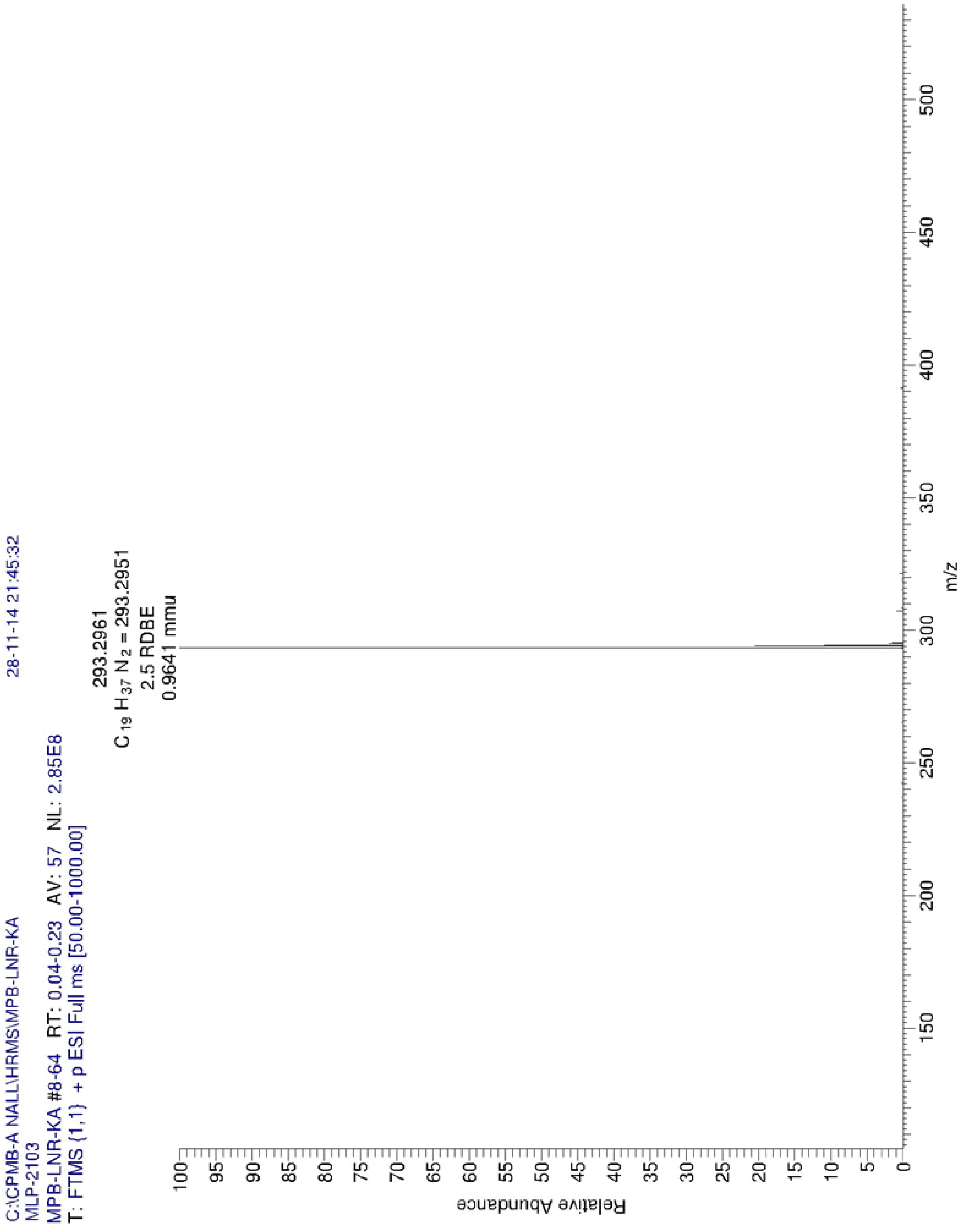

HRMS spectrum of 1-hexadecyl-1H-imidazole

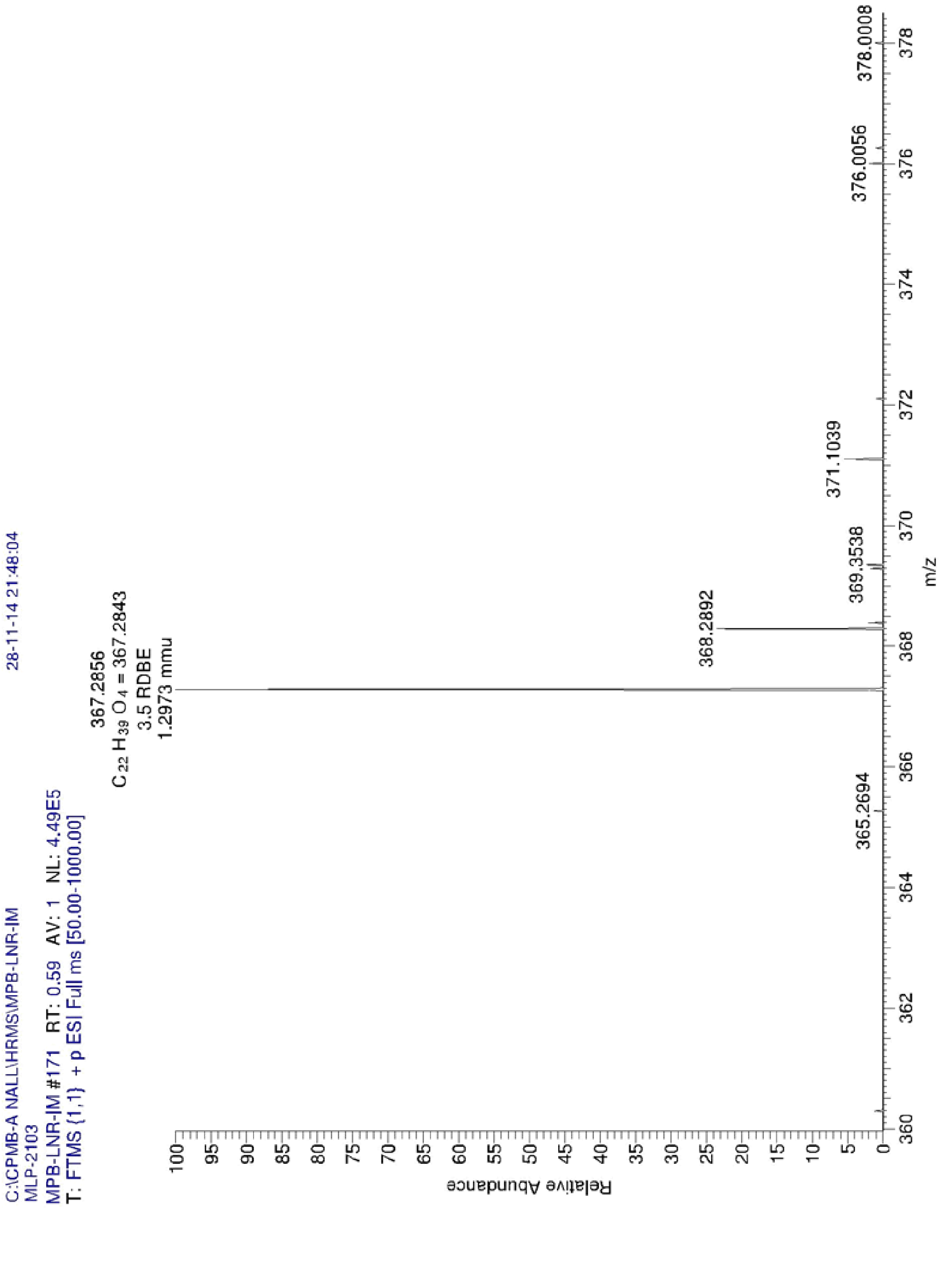

HRMS spectrum of 5-(hexadecyloxy)-2-(hydroxymethyl)-4H-pyran-4-one

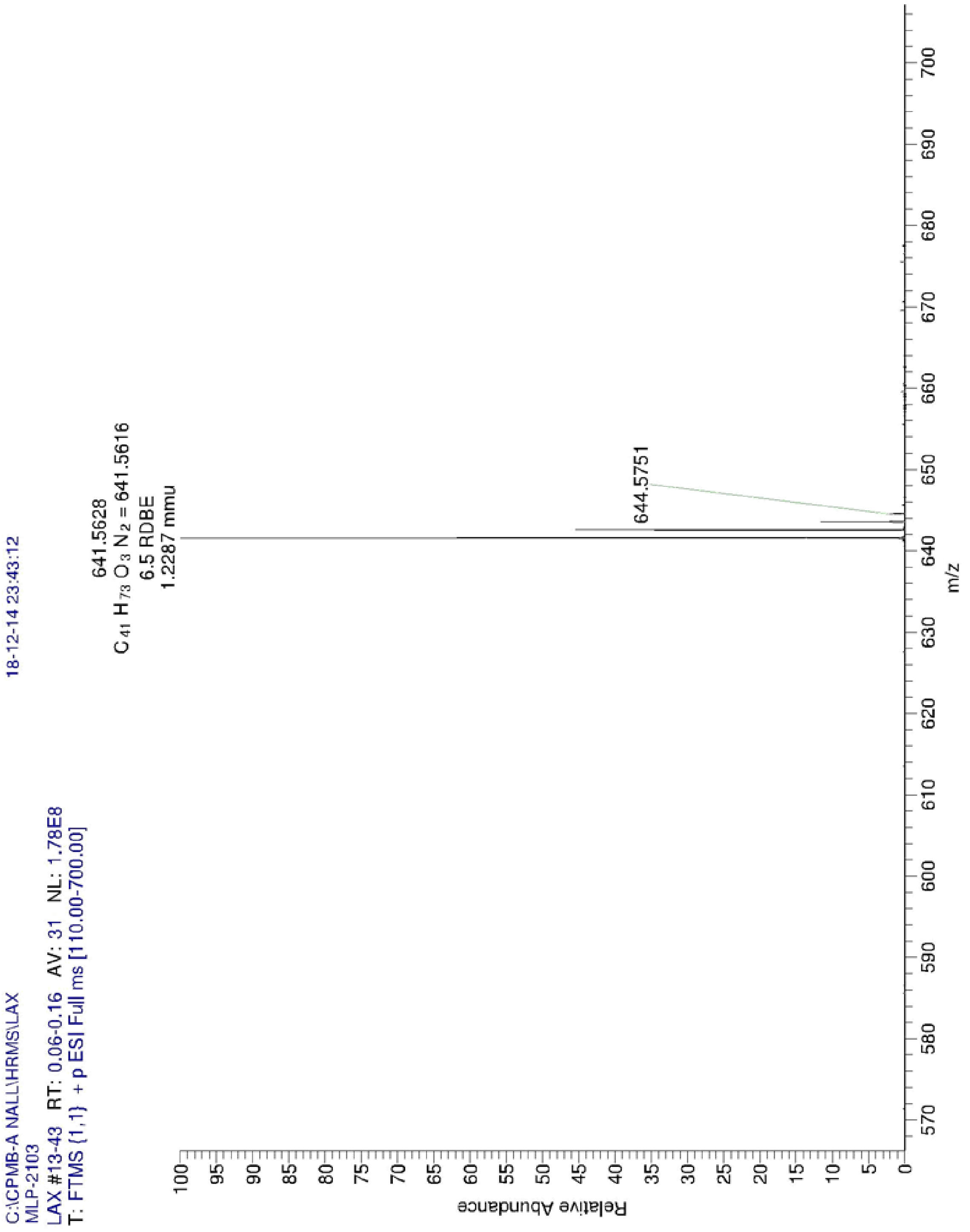

HRMS spectrum of 1-hexadecyl-3-((5-(hexadecyloxy)-4-oxo-4H-pyran-2-yl)methyl)-1H-imidazol-3-ium

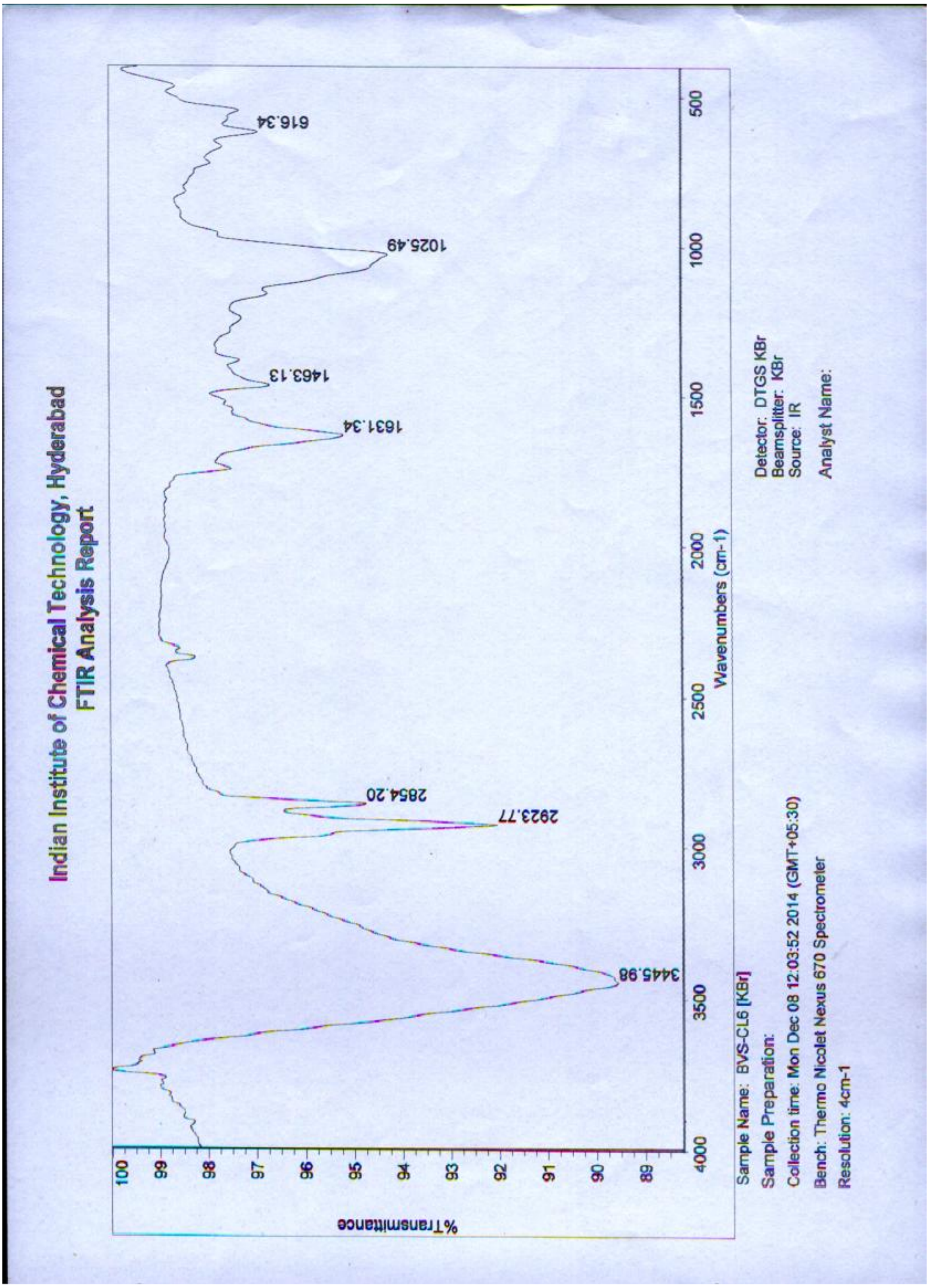

FTIR spectrum of 1-hexadecyl-1H-imidazole

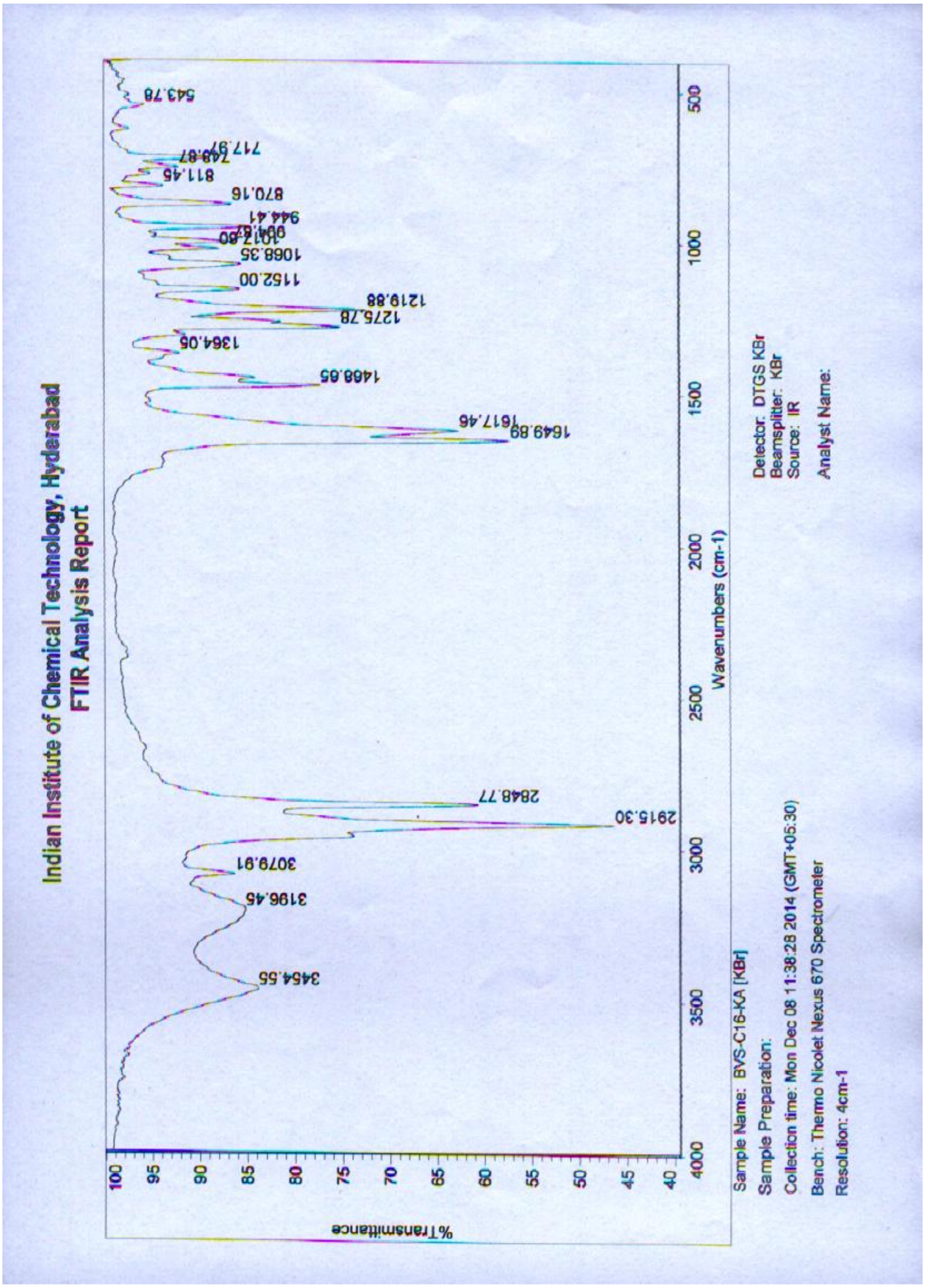

FTIR spectrum of 5-(hexadecyloxy)-2-(hydroxymethyl)-4H-pyran-4-one

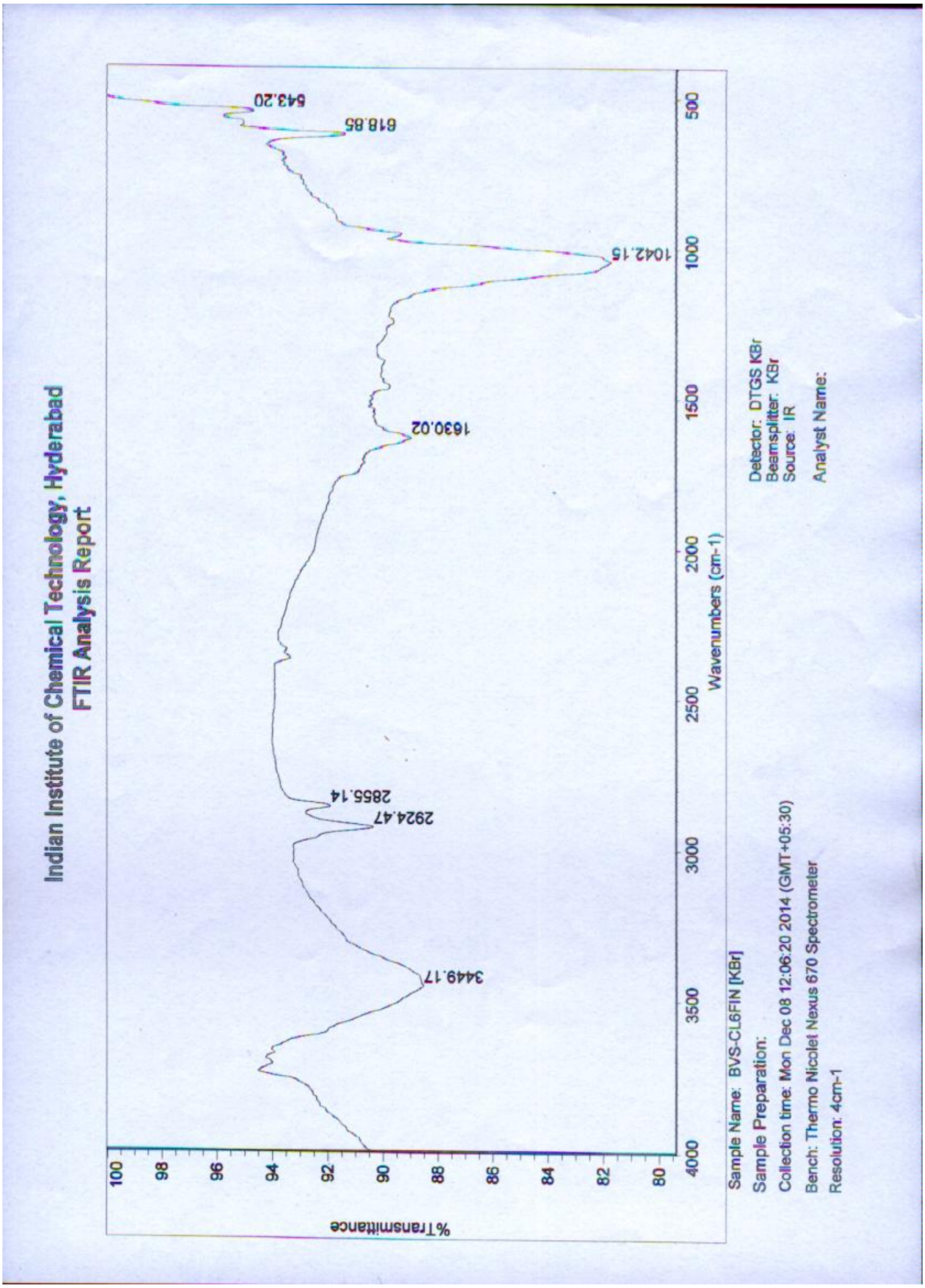

FTIR spectra of 1-hexadecyl-3-((5-(hexadecyloxy)-4-oxo-4H-pyran-2-yl)methyl)-1H-imidazol-3-ium

EXCTRACTED CHROMATOGRAM METHOD REPORT :

Extracted PDA Method : at WL = 272.000 +/- 4.000 nm

User ID : K.HARI
Acquired Time : 4/4/2013 8:53:14 PM
Method Name : NOT DEFINED
System Name : System1

Acquisition :
Description :
SAMPLE NAME:Blank
COLUMN:Hypersil BDS, C18,150x 4.6mm, 5μ,
Mobile Phase : A: ACN, B: 0.1% TFA in Water.
Flow Rate: 1.0 ml/min
Program:
T(min) : 0.01, 15.0 , 35.0
B(%) : 95 10, 10, .
Sample preparation : MeOH
Injection volume : 5.00 μL

Sample mass : 0.00

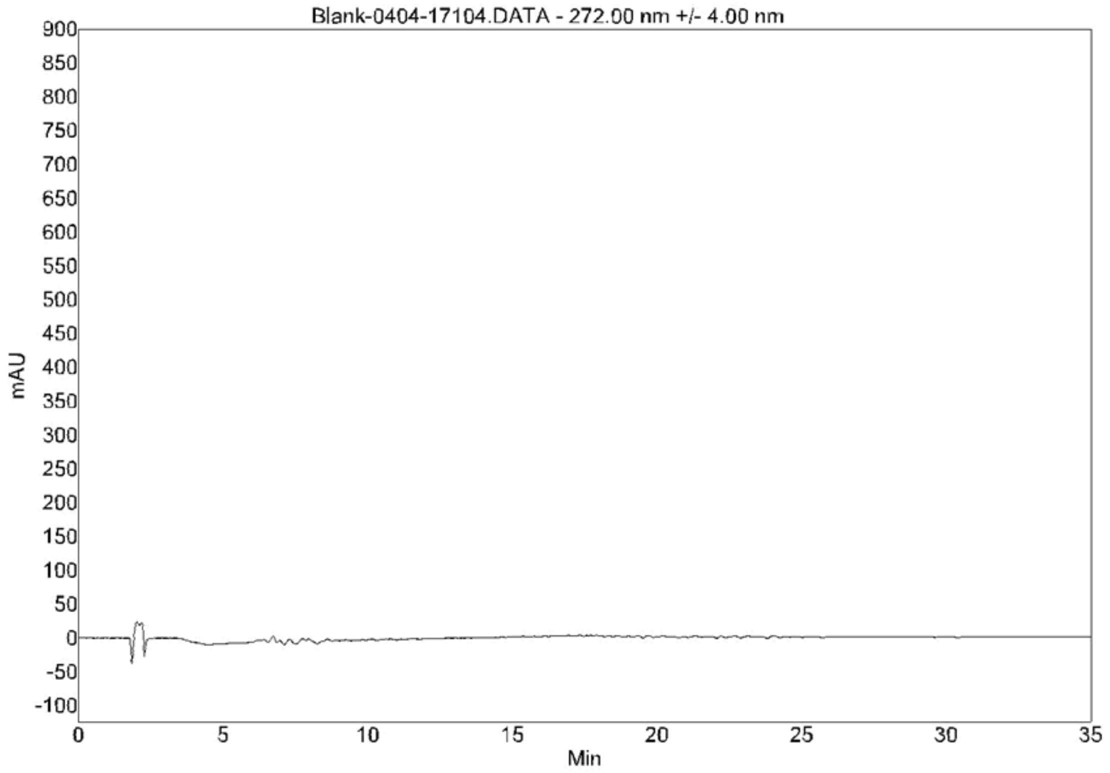

Index	Time [Min]	Area [mAU.Sec]	Res. USP	NTP USP	Area % [%]
Total		0.0			0.000

HPLC Chromatogram of Blank sample

EXCTRACTED CHROMATOGRAM METHOD REPORT :

Extracted PDA Method : at WL = 272.000 +/- 4.000 nm

User ID : K.HARI
Acquired Time : 4/4/2013 10:18:07 PM
Method Name : NOT DEFINED
System Name : System1

Acquisition :
Description :
SAMPLE NAME:16ACID-B
COLUMN:Hypersil BDS, C18,150x 4.6mm, 5μ,
Mobile Phase : A: ACN, B: 0.1% TFA in Water.
Flow Rate: 1.0 ml/min
Program:
T(min) : 0.01, 15.0 , 35.0
B(%) : 95 10, 10, .
Sample preparation : 0.5mg/ml in MeOH
Injection volume : 5.00 μL

Sample mass : 0.00

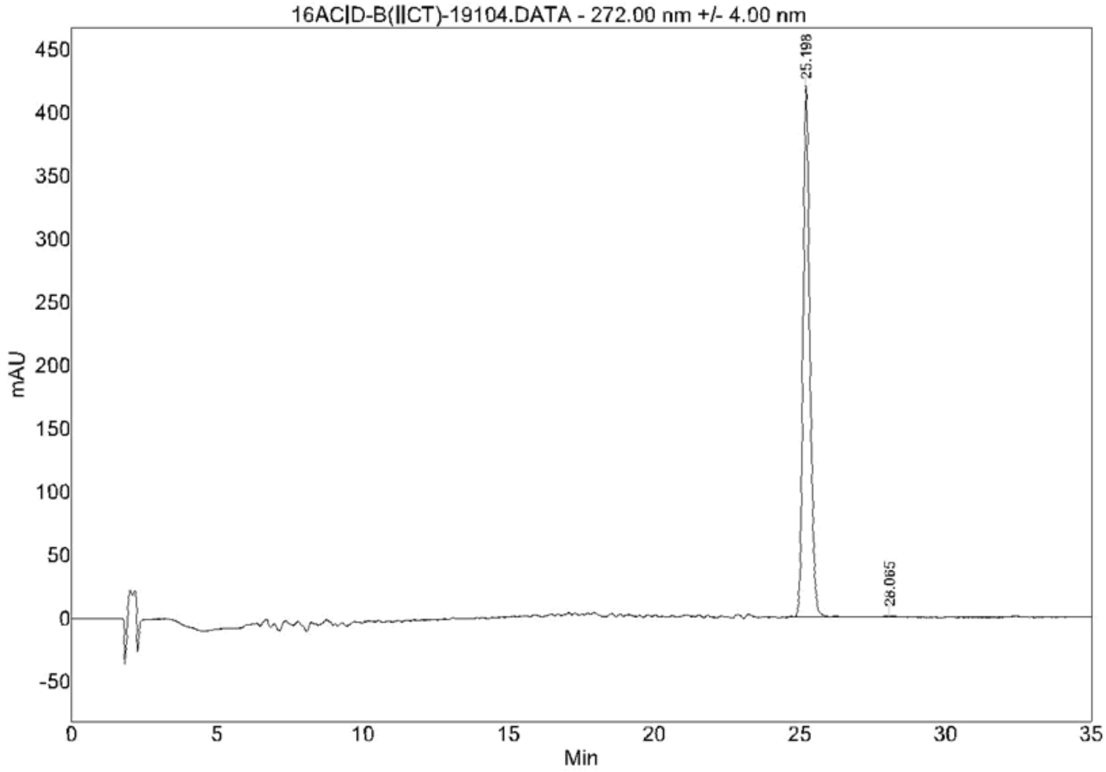

Index	Time [Min]	Area [mAU.Sec]	Res. USP	NTP USP	Area % [%]
1	25.198	6891.8	0.00	55764.29	99.762
2	28.065	16.4	5.09	25742.32	0.238
Total		6908.2			100.000

HPLC Chromatogram of 1-hexadecyl-3-((5-(hexadecyloxy)-4-oxo-4H-pyran-2-yl)methyl)-1H-imidazol-3-iuma
